# NiFeO_x_ decorated Ge-hematite/perovskite for an efficient water splitting system

**DOI:** 10.1038/s41467-021-24428-7

**Published:** 2021-07-14

**Authors:** Ki-Yong Yoon, Juhyung Park, Minsu Jung, Sang-Geun Ji, Hosik Lee, Ji Hui Seo, Myung-Jun Kwak, Sang Il Seok, Jun Hee Lee, Ji-Hyun Jang

**Affiliations:** 1 School of Energy and Chemical Engineering, Department of Energy Engineering, Ulsan National Institute of Science and Technology (UNIST), Ulsan, Republic of Korea; 2 School of Chemical and Environmental Engineering, Dong-Eui University, Busan, Republic of Korea

**Keywords:** Photocatalysis, Artificial photosynthesis, Photocatalysis, Nanoscale materials

## Abstract

To boost the photoelectrochemical water oxidation performance of hematite photoanodes, high temperature annealing has been widely applied to enhance crystallinity, to improve the interface between the hematite-substrate interface, and to introduce tin-dopants from the substrate. However, when using additional dopants, the interaction between the unintentional tin and intentional dopant is poorly understood. Here, using germanium, we investigate how tin diffusion affects overall photoelectrochemical performance in germanium:tin co-doped systems. After revealing that germanium is a better dopant than tin, we develop a facile germanium-doping method which suppresses tin diffusion from the fluorine doped tin oxide substrate, significantly improving hematite performance. The NiFeO_x_@Ge-PH photoanode shows a photocurrent density of 4.6 mA cm^−2^ at 1.23 V_RHE_ with a low turn-on voltage. After combining with a perovskite solar cell, our tandem system achieves 4.8% solar-to-hydrogen conversion efficiency (3.9 mA cm^−2^ in NiFeO_x_@Ge-PH/perovskite solar water splitting system). Our work provides important insights on a promising diagnostic tool for future co-doping system design.

## Abstract (web summary)

Germanium (Ge) has potential as a dopant suitable for the hematite-based photoelectrochemical water splitting system. Here, the authors report the fabrication of Ge doped porous hematite and demonstrate an efficient tandem system of Ge doped porous hematite and the perovskite solar cell.

## Introduction

Hematite (α-Fe_2_O_3_) is considered a promising material for photoelectrochemical water splitting because of its suitable band gap (2.0–2.2 eV), low cost, natural abundance, and good stability in alkaline environments^1–6^. However, hematite features a short hole-diffusion-length (>5 nm)^7–9^, low electrical conductivity^10–12^, and low absorption coefficient issues^1,13,14^. For these reasons, hematite has shown poor oxygen evolution activity as a photoanode, which is strongly related to a high recombination-rate^15–17^. It thus has a substantially lower solar-to-hydrogen (STH) conversion efficiency than the theoretical value (~15%). Doping to improve the poor charge transfer behavior is one of the most powerful strategies that has been suggested to address these notable drawbacks^10,18–25^. But even when Si^22,23,26,27^, Sn^18,19,28–30^ and Ti^20,21,31–36^ atoms were broadly utilized as representative dopants, the hematite photoanode doped with these heteroatoms still exhibited low conversion efficiency.

Therefore, seeking alternative dopants may provide a more straightforward way of overcoming hematite’s low conversion efficiency. Among various dopant candidates^37–39^, germanium (Ge) may be the most promising alternative as an n-type dopant^37^. Ge can dramatically enhance donor density while maintaining the crystallinity of hematite, leading to an outstanding solubility in hematite^37,40^. Prezhdo et al. reported density functional theory (DFT) calculation results showing that Ge was more soluble in hematite than Si and Sn due to the balance between atomic radius and formation enthalpy^37^. Further, Ge has a guiding effect on the preferential growth of the (110) plane of the hematite crystal, which promotes high electrical conductivity^40,41^.

Despite theoretical results that Ge could provide the superior photoelectrochemical properties compared with the current representative dopants in various respects, the highest water splitting performance for Ge-doped hematite reported so far is still far lower than those for representative dopants-doped hematite^18,23,31,40^. The strong discrepancy between calculated results and experimental data for doped hematite may be attributed to some variables that were neglected in the calculation. We hypothesized that unintentional Sn-doping from the fluorine-doped tin oxide (FTO) substrate, which inevitably occurs during the high-temperature annealing process (above 700 ^o^C) is one of such variables. It has been proved that thermal diffusion of Sn from the FTO substrate indeed occurs and it is one of key factors which boosts PEC performance^18,42–44^. Like this, Sn doping from the FTO substrate is known to improve the crystallinity of hematite or the presence of diffused Sn was often ignored in heteroatom-doped systems. Therefore, for a more realistic experimental approach, the presence of the Sn dopant and its specific impact need to be carefully considered in relation with the desired dopant.

Here we report that the water splitting performance of Ge-doped porous hematite (Ge-PH) can bring the experimental data more closely in line with the superior theoretical results of Ge-doped hematite by preventing the unintentional Sn-doping. The approach produces a remarkable performance improvement compared to previous Ge-doped hematite (Ge-H), as well as hematite prepared with the commonly used representative dopants (Ti, Sn, and Si).

We confirmed by both experiment and DFT calculation that when the Ge and Sn dopants were co-present, the crystallinity of the hematite significantly deteriorated due to structural distortion. We also proved that Ge-doping by the thermal diffusion of Ge in the GeO_2_ overlayer, reported in this study, mitigated the Sn diffusion into the hematite lattice and created numerous OER active sites, while maintaining the crystallinity of the hematite surface. More importantly, we report that Ge-PH can lower the overpotential of OER than pure hematite, using both theoretical simulations and experimental data.

With these synergies, our Ge-PH with NiFeO_*x*_ co-catalyst (NiFeO_*x*_@Ge-PH) exhibited a photocurrent density of 4.6 mA cm^−2^ at 1.23 V_RHE_, achieving around a 460% enhancement in PEC performance compared with undoped hematite (~1.0 mA cm^−2^ at 1.23 V_RHE_).

By coupling a perovskite solar cell (PSC) to the back of our photoanode, we achieved ca. 4.8% SHT efficiency for a tandem PEC water splitting system. Our Ge-PH effectively maximized the efficiency of a solar water splitting, supported by a low turn-on voltage system with high performance.

To the best of our knowledge, this work demonstrates the highest STH efficiency for a single hematite photoanode-based tandem device, which may be a stepping-stone for a breakthrough in stagnant hematite-based PEC performance.

## Results and discussion

### Fabrication process and morphology effect

#### Fabrication process of Fe_2_O_3_, Ge-H, and Ge-PH

**Fig. 1:**
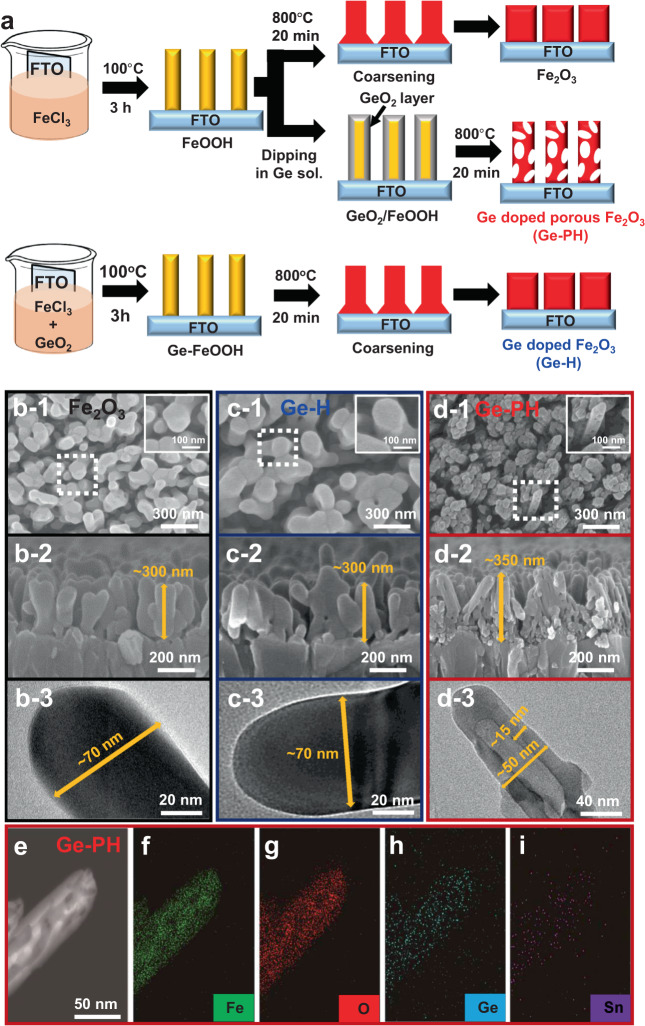
Fabrication process and morphology of Ge-PH. **a** Scheme for fabrication of the Fe_2_O_3_, Ge-H, Ge-PH. **b** Scanning electron microscopy (SEM) and transmission electron microscopy (TEM) images (top-view (b-1, c-1 and d-1), cross-section (b-2, c-2 and d-2) and TEM image (b-3, c-3 and d-3)) of **b** Fe_2_O_3_, **c** Ge-H and **d** Ge-PH. **e** Scanning transmission electron microscopy (STEM) image of Ge-PH and the corresponding elemental mapping of **f** Fe, **g** O, **h** Ge and **i** Sn.

Pristine α-Fe_2_O_3_ (Fe_2_O_3_) and Ge-doped Fe_2_O_3_ (Ge-H) photoanodes were fabricated using conventional methods as reported (Fig. 1a)^40,41,45^. Briefly, β-FeOOH nanorods were grown on an FTO substrate using the common hydrothermal method and then rapidly annealed at 800 ^o^C for 20 min to form Fe_2_O_3_ (top in Fig. 1a). Ge-H (bottom in Fig. 1a) refers to bulk Ge-doped Fe_2_O_3_ hydrothermally grown in a mixture solution of FeCl_3_ and GeO_2_ followed by a rapid annealing step at 800 ^o^C for 20 min as reported previously^5,31^. To fabricate the Ge-PH (middle in Fig. 1a), as-fabricated β-FeOOH nanorods were immersed in a Ge solution for 30 min and rapidly annealed. The Ge solution for doping was made by dissolving GeO_2_ powers in deionized water. Since all of the samples were subjected to the high-temperature annealing step (800 ^o^C for 20 min), which creates Sn-doped Fe_2_O_3_, we deliberately omit mentioning the Sn for simplicity in this study.

#### Morphology of Fe_2_O_3_, Ge-H, and Ge-PH

Figure 1b–d is SEM and TEM images of the as-prepared hematite photoanodes, verifying morphology can be controlled simply by the surface treatment of the β-FeOOH nanorods. Fe_2_O_3_ and Ge-H showed a conventional worm-like morphology with ~70 nm diameter and ~300 nm length. The Fe_2_O_3_ nanorods in the Fe_2_O_3_ and Ge-H had a thicker diameter and lower length than β-FeOOH nanorods, since the Fe_2_O_3_ nanorods were collapsed and coarsened during the high-temperature annealing process, as shown in Fig. 1b, c. However, in Ge-PH, the nanorods had a diameter and length (diameter: ~50 nm and length: ~350 nm) similar to β-FeOOH nanorods, but with a nanoporous structure, as shown in Fig. 1d and Supplementary Fig. 1. By applying a GeO_2_ overlayer on β-FeOOH before the annealing process, nano-porous hematite was created. In brief, the pore generation mechanism in Ge-PH is as follows. (i) The immersion of β-FeOOH into the Ge solution creates a thin GeO_2_ layer on the surface of the FeOOH. The thin GeO_2_ layer serves as a hard template to prevent the melting of the hematite during the annealing process (Supplementary Figs. 2 and 3)^42^. (ii) The GeO_2_/β-FeOOH undergoes in situ conversion into Ge-PH by subsequent high-temperature annealing. In this process, the mass evaporation of water in the hard template generated vacancies in the GeO_2_/β-FeOOH. This high-temperature dehydration creates Ge-PH with mesopores inside, via the previously reported gas entrapping mechanism^5^. In addition, the Kirkendall effect, the motion of the interface between two materials due to different diffusion rates of each atom, was partly involved in the creation of pores, as reported by Gong’s group^46^. Due to the low density of the active material, Ge-PH is more transparent than Fe_2_O_3_ and Ge-H (Supplementary Fig. 4).

As shown in Fig. 1e–i, the energy dispersive X-ray (EDX) mapping of Fe, O, Ge and Sn elements by STEM analysis shows spatially uniform distribution and the porous morphology of the Ge-PH.

Ge-PH with a nanoporous structure has two main advantages over Fe_2_O_3_ or Ge-H. First, the path distance for the generated holes to travel from inside to the surface of the hematite, where oxygen generation occurs, is shortened (10–15 nm), which helps address the critical issues of the short hole-diffusion length of hematite, as shown in Fig. 1d. We compared the PEC performance of the samples with front and back illumination, which confirmed that Ge-PH had the shorter diffusion length for holes to reach the surface as shown in Supplementary Fig. 5. Second, the occurrence of pores in the Ge-PH increases the number of reaction sites for oxygen evolution, simply by increasing the surface area. As shown in the BET data for the surface area and pore distributions (Supplementary Figs. 6 and 7), Ge-PH exhibited five-fold (~10 m^3^/g) increased surface area compared to Fe_2_O_3_ or Ge-H (~2 m^3^/g) with a mesopore morphology. Besides the structural differences between Ge-H and Ge-PH, the Ge in Ge-PH was doped in the final step by the thermal diffusion of Ge from the surface, whereas the Ge was uniformly doped in Ge-H at the beginning step, during the process of forming the β-FeOOH state.

### PEC water oxidation activity and characterization

**Fig. 2:**
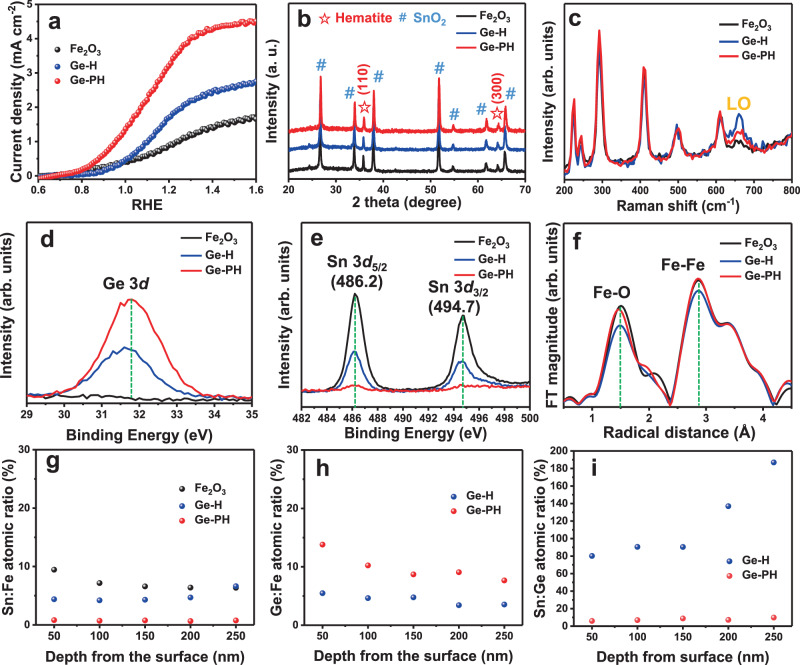
The performance and structure analysis of various photoanodes. **a** Comparison of the photoelectrochemical performance of Fe_2_O_3_, Ge-H, and Ge-PH in a 1 M NaOH (pH = 13.6) electrolyte under simulated sunlight illumination (1 SUN). **b** Comparison of b) XRD patterns and **c** Raman spectra of Fe_2_O_3_, Ge-H, and Ge-PH. XPS spectra of **d** Ge 3d, and **e** Sn 3d. **f** Fourier transform of the EXAFS data at the Fe k-edge of the hematite nanostructures in Fe_2_O_3_, Ge-H, and Ge-PH. The XPS depth profiles of **g** Sn, **h** Ge, and **i** calculated Sn:Ge doping ratio.

Figure 2a compares the photocurrent density generated from samples prepared with different doping methods during the photoelectrochemical water-splitting process. The Ge-PH photoanode delivered around 3.5 times (~3.5 mA cm^−2^ at 1.23V_RHE_) and 1.8 times higher photocurrent density compared to the Fe_2_O_3_ (~1.0 mA cm^−2^ at 1.23V_RHE_) and Ge-H (~1.9 mA cm^−2^ at 1.23V_RHE_) photoanodes, respectively.

To determine whether this remarkable improvement was simply due to the hematite porosity, we fabricated Fe_2_O_3_ with a similar porous morphology using other currently representative dopants (Sn, Ti, or Si). The results clearly showed that Ge was superior to Sn, Ti or Si dopants for hematite, as shown in Supplementary Fig. 8.

The reason can be explained by the advantage of Ge as a dopant in hematite, including the feasible atomic radius of Ge, and the low formation enthalpy of the secondary phase of GeO_2_ as previously reported^27^.

In particular, Ge-PH showed high performance at low voltage without an anodic shift in the onset potential, despite the doping. It has been reported that, in a typical doping system, the increase in defect sites produced by doping can enhance carrier mobility, and carrier density in the bulk, while simultaneously providing recombination sites on the surface, resulting in an anodic shift of the onset potential^47^. Furthermore, the Fe^2+^ formed by n-doping in hematite is also known to act as a recombination site on the surface, which consequently retards the water oxidation reaction in doped hematite^48^. This is in accordance with the anodic shift of the onset potential for all doped hematite photoanodes (Sn, Ti, Si-doped hematite), including the Ge-H in this study, compared to that for Fe_2_O_3_. Therefore, one of the critical issues has been to optimize the two components (onset potential vs current density), which operate in an opposite manner at 1.23V_RHE_.

Thus, the result here that the Ge-doping in Ge-PH did show a cathodic shift of the onset potential is certainly interesting (Supplementary Fig. 9), and suggests that there could be an important factor in our Ge-PH (Surface Ge-doped hematite).

To explore this phenomenon more systematically, we carried out various scientific analyses of Ge-H and Ge-PH. First, the XRD patterns showed similar hematite peaks to Fe_2_O_3_ without the new phase formation in Ge-H and Ge-PH, as shown in Fig. 2b. In the Raman spectra, the appearance of the forbidden longitudinal optical (LO) mode, corresponding to the peak at 660 cm^−1^, is indicative of the symmetry breakdown induced by structural disorder, scattered LO phonons^4^. The LO peak was largely increased and broadened in the Ge-H, compared to Fe_2_O_3_, whereas the much reduced LO peak was observed in Ge-PH, as shown in Fig. 2c. This implies the symmetry breaking by Ge-doping in Ge-H is much larger than in Ge-PH.

A correlation between the Ge and Sn dopants was confirmed by X-ray photoelectron spectroscopy (XPS) data. The observation of a Ge 3d peak at ~31.6 eV from Ge-PH indicates that the Ge atoms were successfully doped in Fe_2_O_3_ with a higher content of Ge compared to Ge-H by the high-temperature annealing process, as shown in Fig. 2d and Supplementary Fig. 10^41,45^.

The Sn 3d peaks centered at 494.7 (Sn 3d_3/2_) and 486.2 eV (Sn 3d_5/2_) of Fe_2_O_3_, Ge-H, and Ge-PH in Fig. 2e suggest that all of the hematite samples were unintentionally doped by Sn^4+^ ions from the FTO substrate during the high-temperature annealing process^18,42^. It should be noted that the Ge-PH had much a lower Sn dopant content than Fe_2_O_3_ and Ge-H. This can be attributed to the GeO_2_ overlayer and the relatively long and thin nanorod morphology of Ge-PH, as compared with the coarsened short and bare nanorods of Fe_2_O_3_ and Ge-H, as shown in Supplementary Fig. 3^42^. The thinner and longer nanorods of the Ge-PH have lower chances of bulk Sn diffusion from the bottom FTO substrate during the high-temperature annealing process. In addition, the GeO_2_ overlayer can suppress surface Sn diffusion from the surrounding area of the nanorods on the FTO substrate.

In order to check the effect of Sn content on the crystallinity of the doped sample, we carried out extended X-ray absorption fine structure (EXAFS) measurements, which also included information about the inter-atomic distance and the local dynamics of the system. X-ray absorption near edge structure (XANES) results which show the oxidation state change of the host atom can be found in Supplementary Fig. 11. The Fourier transform of the EXAFS results in the R space in Fig. 2f shows a clear difference among the three samples with different doping conditions. The peak around 1.5 Å and 3 Å can be attributed to Fe–O bonds and Fe–Fe bonds, respectively. The decreased intensity of the Fe–O and Fe–Fe bonding lengths for Ge-H compared to Fe_2_O_3_ was confirmed, revealing there was a prominent distortion of the crystal structure after Ge doping. Also, an increased R space was observed for the Fe–O bonding length in Fe_2_O_3_ and Ge-H, indicating the formation of a lower oxidation state of Fe, such as Fe^2+^.

We hypothesized that these probably suggest that Sn has a greater influence on structural distortion, due to the larger atomic size than Fe and the excess charge coming from the n-type dopants^49^.

**Fig. 3:**
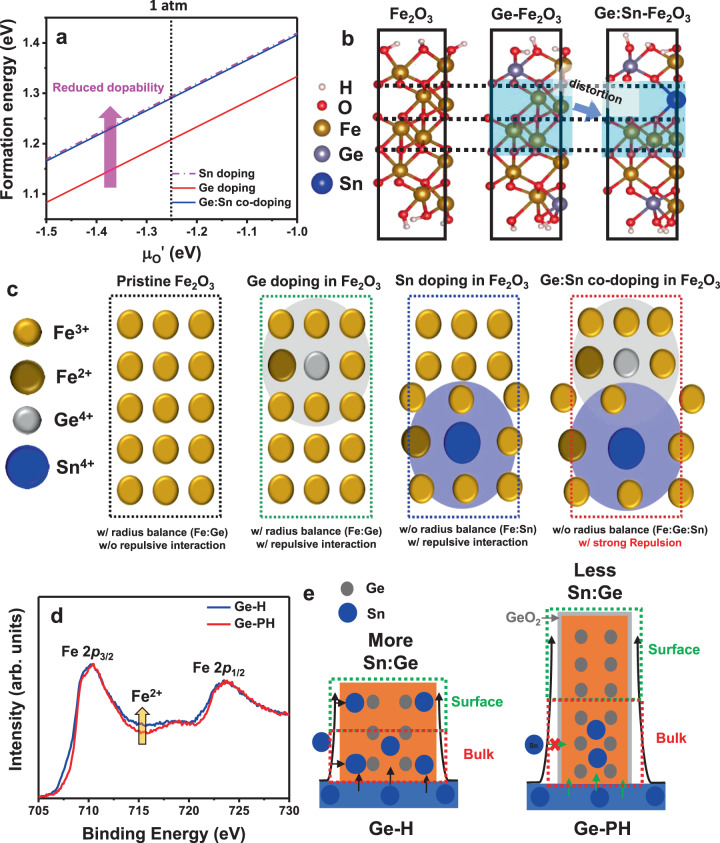
DFT calculations and experimental results for Ge and Sn interaction. **a** The formation energy for Sn-doping, Ge-doping, and Ge:Sn co-doping in Fe_2_O_3_. The dotted line represents 1 atm at which the experiments were performed. **b** DFT calculations of atomic arrangements of Fe_2_O_3_ with different dopant environments. **c** Atomic structure of Ge-doped Fe_2_O_3_ Sn-doped Fe_2_O_3_ and Ge:Sn co-doped Fe_2_O_3_. **d** XPS spectra of Fe 2_p_. **e** Schematics of the distribution of Sn and Ge according to co-doping methods.

To clearly pinpoint these assumptions, the chemical compositions of Fe_2_O_3_, Ge-H, and Ge-PH were examined by XPS depth profile. The results showed that the Sn-doping ratio of Fe_2_O_3_ and Ge-H were 4.5–9.5% in the whole region. However, the doping ratio of Sn in Ge-PH was much reduced, with a maximum 0.7–0.8% in the whole region as shown in Fig. 2g. This suggests that unintentional Sn-doping by thermal diffusion from the FTO substrate was suppressed by the GeO_2_ overlayer in the long and thin nanorods compared to the short and thick nanorods without the overlayer. Since Ge-PH has an unfavorable and long Sn diffusion path from the bottom FTO substrate, it has less Sn content on the surface of the hematite where the OER reaction occurs, resulting in fewer chances for Ge:Sn combination, as described in Fig. 3e.

The Ge-doping ratios of Ge-H and Ge-PH were measured to be 3.4–5.5% and 7.7–13.8% in the whole structure region, as shown in Fig. 2h. Although the total doping content (Sn + Ge) of Ge-H and Ge-PH was similar (around 8–14%, Supplementary Figs. 12 and 13), the doping ratio (Sn/Ge) of Ge-H was 14–19 times higher than in Ge-PH. Therefore, we can conclude that when the content of Sn increases significantly, it will have a negative effect that causes structural distortion (as proven in Fig. 2c, f). The lower content of Sn:Ge in the surface region of Ge-PH, which was clearly observed in the XPS depth profile, well explains the cathodic shift of the onset potential, indicating reduced recombination in the OER reaction. We checked the content of the oxygen vacancy in each sample because it can promote the reaction kinetic and increase the carrier density to enhance the charge transfer and suppress recombination. Ge-PH showed the highest oxygen vacancy content as shown in Supplementary Fig. 14. Since the oxygen vacancy content increases with an increasing doping concentration and Ge is easily diffused from the surface GeO_2_ layer, the oxygen vacancy of Ge-PH was high but still within the oxygen vacancy level of typical Fe_2_O_3_^50^. As can be seen in Supplementary Fig. 14b, all samples showed similar OER curves in dark conditions which indicate that the level of the oxygen vacancy contained in our samples did not cause a change in the OER mechanism.

### Theoretical and experimental investigation on the effect of Ge:Sn co-doping

#### Solubility of Sn and Ge in hematite

To understand the solubility of Sn and Ge in hematite, we calculated the formation energy for Sn-doped Fe_2_O_3_, Ge-doped Fe_2_O_3,_ and Ge:Sn co-doped Fe_2_O_3_, as shown in Fig. 3a. The formation energy for Ge:Sn co-doping (blue) and Sn-doping (pink) was higher than that for Ge-doping (red). The high formation energy of Ge:Sn co-doping in Fe_2_O_3_ specifically indicates the low dopant solubility and low ionization of the Ge dopant in hematite^37^. We explored the structure distortion caused by the presence of Ge and Sn by comparing the atomic structures of Ge-doped hematite and Ge:Sn co-doped hematite using DFT calculations. As can be seen in Fig. 3b, the Ge:Sn co-doped hematite experiences greater symmetry breaking after the re-positioning of the Fe atoms, while the substitutional single Ge-doping did not produce any noticeable distortion in the atomic arrangement.

Based on DFT calculations, we drew the atomic arrangement of hematite with the substitution of heteroatoms to clearly understand this phenomenon, as shown in Fig. 3c. Single-Ge-doped hematite did not show much distortion since the Ge dopant has a radius similar to Fe in hematite, and Ge becomes more soluble than other representative metal dopants. In the case of Sn-doped hematite, relatively high structural distortion occurs since the Sn dopant has a larger radius than Fe. When Ge and Sn are co-present, additional strong electron repulsion between Fe atoms neighboring the Ge and Sn dopants is produced by the excess electron charges from the n-type metal dopants in Fe_2_O_3_. FTO is preferentially used as a substrate of hematite-based photoelectrodes, since it withstands the high temperatures for hematite activation (>700 ^o^C) relatively well compared to other transparent substrates, including AZO or ITO^43,51,52^.

#### Observation of the structural disorder

Figure 3d is the XPS spectra of Fe 2_p_, which shows that Ge-H has more Fe^2+^ than Ge-PH. A structural strain can occur due to a mismatch in the atomic radius upon doping. Sn has a very large radius compared to Fe, whereas the Ge atom has a similar radius to the Fe atom. Since Fe_2_O_3_ and Ge-H have more Sn content compared to Ge-PH, a higher content of Fe(II) was observed as shown in Fig. 2g. Ge-H has a large amount of diffused Sn, where Ge is present throughout the hematite nanorod. In Ge-PH, however, the majority of the Ge and Sn dopants are positioned in different regions, and the amount of diffused Sn is relatively small in the surface region where the OER reaction occurs specifically, thus minimizing the adverse effect caused by the co-existence of the two n-dopants, as shown in Fig. 3e. Therefore, due to the lower content of Sn, Ge-PH was expected to experience a lesser distortion than Ge-PH.

These results well explain the XRD, Raman, EXAFS, XPS spectra, onset potential, and the PEC activity observed in Fig. 2 and Supplementary Fig. 9, which show that the structural distortion observed in Ge-H caused by co-doping of Sn and Ge in hematite was almost recovered in Ge-PH, which had a status similar to the original undoped hematite.

### Recombination rate and surface activity

**Fig. 4:**
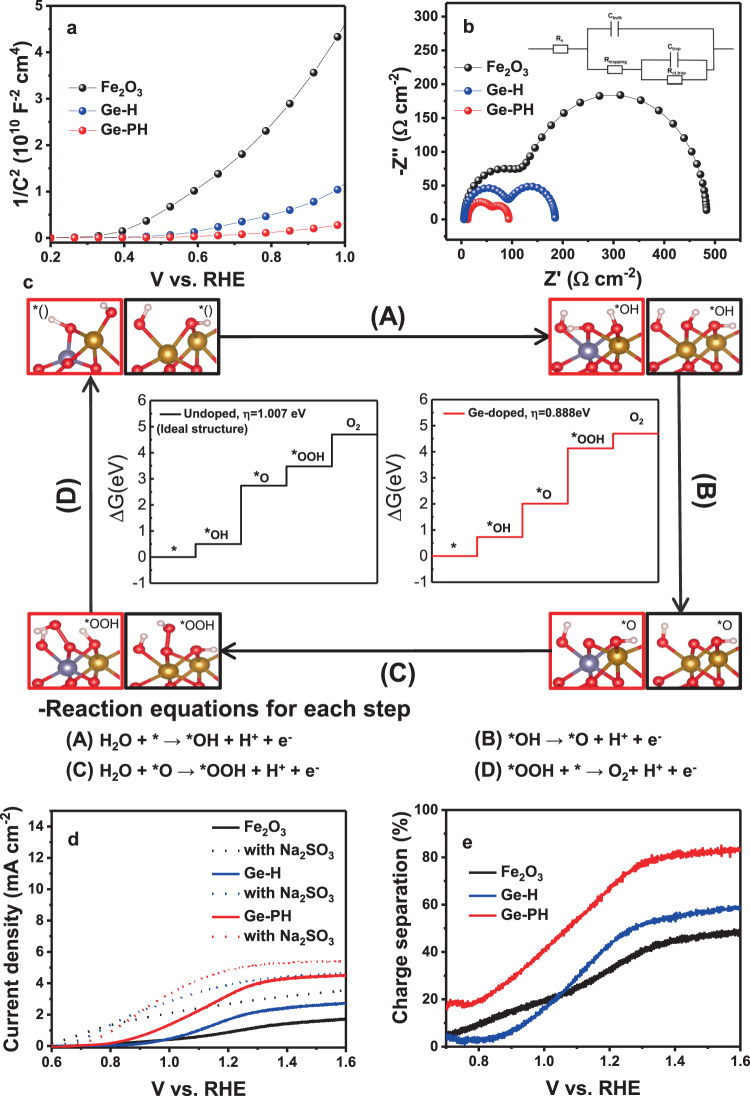
Electrochemical analysis and simulated OER activity. **a** Mott-Schottky plots and **b** EIS measurements (with the circuit model). **c** Free energy diagrams of the intermediates on Fe_2_O_3_ (undoped) and Ge-Fe_2_O_3_ (Ge-doped). For comparison, the free energies of the OER species on the hematite (0001) surface from the literature are included. Brown, gray, red, and white are Fe, Ge, O, and H atoms, respectively. **d** LSV curves of Fe_2_O_3_, Ge-H, and Ge-PH under illumination in 1 M NaOH (solid line) and a solution mixture of 1 M NaOH and 0.5 M Na_2_SO_3_ (dashed lines). **e** Charge separation efficiencies.

Between the two n-type elements investigated in this study, Ge is superior to Sn, which was confirmed by electrochemical analysis (Supplementary Fig. 15). The highest charge carrier density of Ge-PH, which is inversely proportional to the lowest slope of the curve in the Mott-Schottky plot (Fig. 4a), was in a good agreement with the simulation and experimental results. These results are consistent with the impedance data in Fig. 4b and electrical conductivity data in Supplementary Fig. 16. Therefore, enhanced carrier density and electrical conductivity of Ge-PH can maximize the PEC performance. From the Mott-Schottky plot, we also investigated the flat band potential as shown in Table 1. Basically, hematite with a low flat band potential delivers a low onset potential as in the previously reported work^53^. In our photoanodes, Fe_2_O_3_ showed the lowest flat band potential (0.39V_RHE_) compared to Ge-H (0.53V_RHE_) and Ge-PH (0.43V_RHE_). However, the similar onset potential of Fe_2_O_3_ and Ge-PH was observed due to the make-up in the flat band potential by the favorable activation energy in Ge-PH.

**Table 1:** Representative results for the flat band potential (EFB), charge carrier concentration (ND), and space charge width (Wsc) from Mott-Schottky measurements.

	E_FB_ (V)	N_D_ (10^20^ cm^−3^)	W (nm)
Fe_2_O_3_	0.39	0.22	12.4
Ge-H	0.53	0.70	6.26
Ge-PH	0.43	3.10	2.97

Figure 4b shows the Nyquist plots used to investigate the influence on the electrolyte/Fe_2_O_3_ interface. The R_ct_ value (charge transfer resistance between the electrolyte/material, second semicircle) of Ge-PH was much smaller than that of Fe_2_O_3_ or Ge-H. And R_trap_, a resistance related to the rate of trapping holes in the surface states (first semicircle), was also smaller than that of the other photoanodes.

To support the reduced overpotential and excellent performance in Ge-PH observed in our experimental result, DFT calculations were performed to determine the theoretical overpotential. Figure 4c shows the calculated free energy for each elementary step. In our limited study, an ideal hematite structure (without unintentional Sn diffusion) was analyzed to confirm the Ge dopant effect on the theoretical OER values (undoped vs. Ge-doped). It is known that the rate-determining step for hematite is the reaction B (*OH → *O) where the deprotonation from *OH can make the charge state (*O) very unstable^54–56^. In undoped hematite, therefore, the reaction B corresponding to deprotonation from *OH has the highest free energy in the reaction pathway and the reaction potential was determined to be 2.2372 eV. The calculated overpotential for undoped hematite is 1.007 eV, which is in reasonable agreement with previous theoretical studies for (0001) hematite^54,55^.

To lower the free energy for the reaction B, it is necessary to reduce the instability of *O. When Ge is doped in hematite, the charge state of *O can be more stable since an n-type dopant Ge provides the electron to oxygen^37,39,54^. Therefore, the free energy of the reaction B is significantly reduced by Ge doping. On the other hand, due to a trade-off relationship of the free energy between the reaction B (*OH → *O) and the reaction C (*O → *OOH)^54^, the rate-determining step of Ge-doped hematite is the reaction C, which has a 0.119 eV lower overpotential (0.888 eV) than undoped hematite, which is in consistent with our experimental J-V curve.

Charge separation efficiency was calculated based on the LSV curves under illumination in 1 M NaOH and 1 M NaOH containing 0.5 M hole scavenger, Na_2_SO_3_, as shown in Fig. 4d. Notably, Ge-PH showed a substantially higher charge transfer efficiency than Fe_2_O_3_ and Ge-H over the entire tested potential range, and approached 80% at potentials beyond 1.3V_RHE_ as shown in Fig. 4e. The results of the electrochemical analysis and DFT calculations clearly support the reason for the low onset potential of Ge-PH.

### NiFeO_*x*_@Ge-PH/PSC solar water splitting performance

In order to confirm the feasibility of our photoanode for solar water splitting, we evaluated the performance of Ge-PH in a tandem configuration^57–61^. We prepared a tandem device containing a single PSC and a hematite photoanode similar to the Z-scheme in natural photosynthesis, in which two semiconductors with different absorption spectra are efficient over a broad part of the solar spectrum, and deliver a high STH efficiency for water splitting.

For this setup, we employed a PSC fabricated using a recently developed procedure (short-circuit current (*J*_sc_) = 21.60 mA cm^−2^, open-circuit voltage (*V*_oc_) = 1.16 V, and fill factor (FF) = 75.07%; power conversion efficiency (PCE) = 18.85%, Supplementary Fig. 17)^62^. The PSC is unable to drive the reaction on its own (or with an efficient electrocatalyst) because its photovoltage is less than what is thermodynamically required to split water^58,59^.

**Fig. 5:**
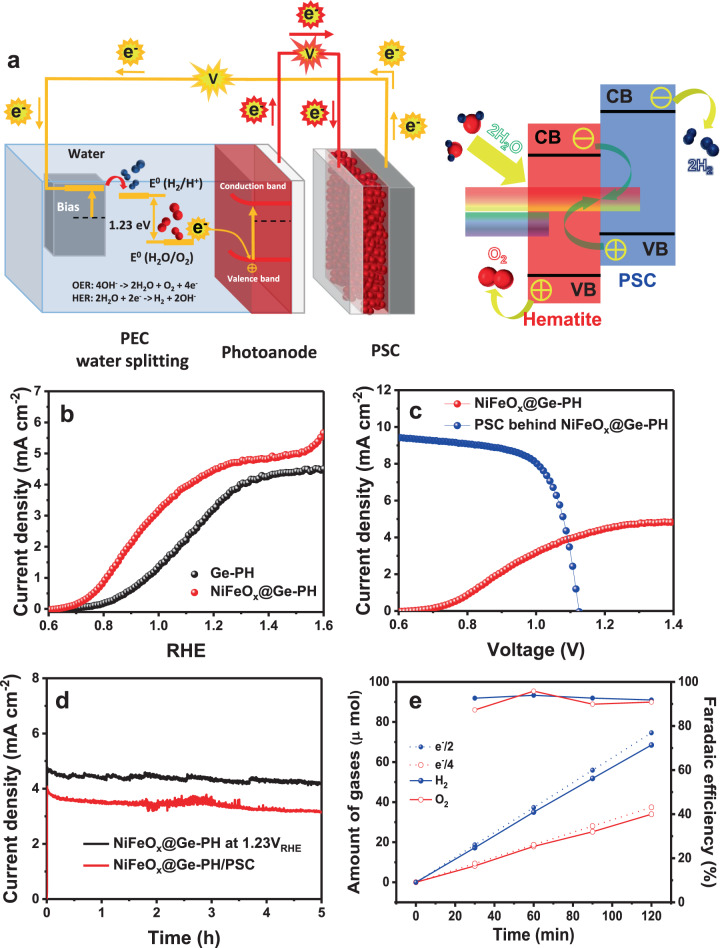
NiFeO_x_@Ge-PH/PSC solar water splitting efficiency. **a** Schematic of the tandem system featuring a perovskite solar cell (PSC) and photoanode with a Z-scheme of artificial photosynthesis driven by light absorption (left). Hematite was used to evolve oxygen and the PSC was used to evolve hydrogen (right). **b** J-V curves of Ge-PH and NiFeO_*x*_ decorated Ge-PH. **c** J-V curves of a PSC-hematite-based photoanode tandem device together. **d** Stability of the NiFeO_*x*_@Ge-PH at 1.23V_RHE_ and NiFeO_*x*_@Ge-PH/PSC solar water splitting. **e** Faradaic efficiency of the NiFeO_*x*_@Ge-PH.

A schematic of the tandem configuration, with the PSC connected electrically and optically in series with the hematite is shown in Fig. 5a. To boost PEC performance, NiFeO_x_, one of the representative OER catalysts used in hematite-based PEC systems, was deposited on the surface of the Ge-PH photoanode. NiFeO_x_ can greatly reduce the recombination via a facile charge separation process by enhancing the transfer kinetics for OER. Therefore, NiFeO_x_/Ge-PH (in water) achieved almost the same performance as Ge-PH with a sacrificial agent (in sulfite). When NiFeO_*x*_ was applied to Ge-PH, the photocurrent density of the NiFeO_*x*_@Ge-PH reached 4.6 mA cm^−2^ at 1.23 V_RHE_ as shown in Fig. 5b. In addition, the NiFeO_x_ catalyst helped shift the onset potential with enhanced performance (Supplementary Fig. 18). When NiFeO_x_ was applied to Ge-PH, the photocurrent density of the NiFeO_*x*_@Ge-PH reached 4.6 mA cm^−2^ at 1.23 V_RHE_ as shown in Fig. 5b.

To estimate the operating current density, J-V curves of the PSC were measured by placing the hematite photoanode before the solar cell to account for optical absorption by the hematite photoanode as shown in Fig. 5c. The operating current density in the tandem configuration was thus estimated to be around 3.9 mA cm^−2^. The assembled tandem device was subsequently tested in 1 M NaOH electrolyte without additional external bias in a two-electrode configuration, using the current density versus time (J-t) curve under 1 SUN (AM 1.5 G, 100 mW cm^−2^). The current density closely matched the operating current extracted from Fig. 5c, with good stability, as shown in Fig. 5d and Supplementary Fig. 19. The STH conversion efficiency was calculated to be 4.8% for the Ge-PH and PSC tandem systems. To the best of our knowledge, this is the highest STH efficiency obtained for a single hematite-based photoanode with a tandem device, as shown in Supplementary Tables 1–3.

Finally, we calculated the faradaic efficiency of the tandem device by measuring the H_2_ and O_2_ evolution under AM 1.5 illumination in 1 M NaOH electrolyte. As shown in Fig. 5e, the hydrogen gases produced on the Pt mesh and the oxygen gases on NiFeO_*x*_@Ge-PH were around 68.5 and 34.0 µmol after 120 min, respectively, indicating a 2:1 ratio of the water splitting mechanism. The ratio between the measured and predicted gas evolution rates gives a faradaic efficiency of 87–95% throughout the measurements. Therefore, most of the photo-generated charges were consumed by water splitting (hydrogen/oxygen gas generation) in our tandem system.

In summary, we present an approach to achieve the theoretically potential results in a water-splitting system of co-doped hematite. We demonstrated that the morphology-controlled Ge-doped hematite with the reduced content of unintentionally doped Sn can be a stepping-stone to approach hematite’s theoretical efficiency, including the high photocurrent density and the low turn-on voltage. Employing our findings and enhanced performance, the NiFeO_*x*_@Ge-PH/PSC tandem system delivered the photocurrent density of ~3.9 mA cm^−2^ in 1 M NaOH electrolyte. Therefore, our insight and co-doping strategy, which reduces the Sn content in hematite for water splitting, potentially provides a paradigm for electrode design and could be further extended to other heteroatom-dopant systems (Ti, Sn, Si, Zr, and Ga) utilized in numerous applications, including solar conversion, sensing, and opto-ferroelectric devices which require doping for enhanced electrical conductivity.

## Methods

### Preparation of the Fe_2_O_3_ and Ge-doped Fe_2_O_3_ (Ge-H) photoanode

Bare Fe_2_O_3_ as a reference photoanode was grown on FTO glass utilizing aqueous chemical growth methods. The synthesis of β-FeOOH rods was performed in an aqueous solution containing 100 mL of 150 mM ferric chloride hexahydrate (FeCl_3_ ∙ 6H_2_O), and Ge-doped β-FeOOH rods was mixed with 500 μl Ge precursor. Ge precursor is completely dissolved in DI water when sonicated with 40 mM GeO_2_ for 6 h to make clean solution. The solution was placed in a cap-sealed glass vial containing two back-to-back slips of FTO glass leaning against the inner wall. The glass vial was placed in a forced convection oven with a programmable temperature controller. After heating to 100 ^o^C from 30 ^o^C for 2 h, the temperature was maintained for 3 h, during which β-FeOOH or Ge-doped β-FeOOH rods were synthesized on the FTO substrate. The sample was thoroughly washed by water and dried by N_2_ gas. The β-FeOOH rods on the FTO substrates were rapidly inserted into a furnace tube at 800 ^o^C for 20 min and taken out to ambient conditions.

### Preparation of the Ge-doped porous Fe_2_O_3_ (Ge-PH) photoanode

β-FeOOH grown on the FTO substrate was immersed in Ge precursor for 30 min. After washing the sample with DI water and drying by N_2_ gas, the GeO_2_/β-FeOOH was annealed using the same method (rapid insertion into a furnace tube at 800 ^o^C for 20 min and removal to ambient conditions) as was used in the preparation of Fe_2_O_3_ or Ge-H to create hematite with Ge-PH.

### Decoration of NiFeO_*x*_ oxygen evolution catalysts on Ge-PH

For depositing the NiFeO_*X*_ OER catalyst on Ge-PH, the Ge-PH photoanode was immersed in a NiFeO_x_ precursor solution. Precursor solutions were prepared from iron(III) 2-ethylhexanoate (50% w/w in mineral spirits) and nickel(II) 2 ethylhexanoate (78% w/w in 2-ethylhexanoic acid) by dissolving the appropriate amount of the metal precursor in hexanes to give a total concentration of a 15% w/w metal complex. These solutions were further diluted with hexane to prepare a solution with a total metal concentration of 50 mM. The photoanode was irradiated with UV light (254 nm) for 1 h and was then annealed in a preheated furnace at 100 ^o^C for 1 h.

### Characterizations

The structures of the samples were characterized by SEM (Nano-SEM 230, 15 kV), TEM (JEM-2100, 200 kV), and Raman spectroscopy (WItec, alpha300R, excited by a 532 nm laser). X-ray diffraction measurements were carried out with a Rigaku Co. High power X-ray Diffractometer D/MAZX 2500 V/PC from 10^o^ to 80^o^).

### PEC measurements

A three-electrode configuration in a homemade photoelectrochemical kit with an exposure area of 0.09  cm^2^ and front-side simulated AM 1.5 illumination (Sol2A class ABA 94062 A, 1000 W Xenon lamp, Newport), composed of an Ag/AgCl (KCl sat.) electrode and a Pt mesh as the reference and counter electrodes, respectively, were used for PEC measurements. The intensity of light was adjusted to 100 mW cm^−2^. A 1.0 M NaOH solution was used as a pH 13.6 electrolyte. Potentials versus RHE were calculated using the Nernst equation *E*_RHE_ = *E*_Ag/AgCl_ + 0.0591(pH) +  0.1976 V. The scan rate for J-V curves was 20 mV s^−1^. EIS was carried out at a frequency range from 100 kHz to 0.1 Hz using a potentiostat (VersaSTAT 3). EIS experimental data were analyzed and fitted using the Z-view software.

### Methylammonium iodide (MAI) synthesis

Methylammonium iodide was synthesized by adding dropwise 57% aqueous hydriodic acid (HI, 30.0 mL) into 40% aqueous methylamine (19.6 mL) solution with vigorous stirring under an ice bath. After the addition of HI, the solution was stirred for another 2 h. The solvent was then removed under vacuum using a rotary evaporator and the precipitate was dissolved in ethanol and recrystallized in diethyl ether. The precipitates were collected by suction filtration and the resulting MAI was then dried at 60 °C under vacuum overnight. Formamidinium iodide (FAI) was also synthesized under identical conditions as described above.

### Synthesis of FAPbI_3_ and MAPbBr_3_ powders

Stoichiometric amounts of FAI and PbI_2_ for FAPbI_3_, MAI, and PbBr_2_ for MAPbBr_3_ were dissolved in 2-methoxyethanol under stirring at 120 °C for 30 min and 100 °C for 30 min, respectively. The precipitates were collected by suction filtration and dried under vacuum overnight.

### Photovoltaic device fabrication

FTO-coated glass (Pilkington, TEC8) as a substrate was cleaned in an ultrasonic bath using detergents, acetone, and ethanol for 30 min, respectively. Titanium diisopropoxide bis(acetylacetonate) precursor solution diluted in ethanol with a ratio of 1:10 by volume was sprayed on the FTO substrate at 450 °C for coating of TiO_2_ hole blocking layer (bl-TiO_2_). Mesoporous TiO_2_ layer (mp-TiO_2_) was then deposited onto the bl-TiO_2_/FTO substrate by spin-coating TiO_2_ paste with an average particle size of 50 nm at 1500 rpm for 50 s. Afterwards, the substrate was annealed at 500 °C in air for 1 h. The synthesized FAPbI_3_ and MAPbBr_3_ powders with 8:2 mole ratio were dissolved in *N*-*N*-dimethylformamide (DMF) and dimethylsulfoxide (DMSO) with the DMF to DMSO volume ratio of 4:1 under stirring at 60 °C for 1 h. The perovskite solution filtered by PTFE syringe filter (0.2 μm) was deposited on the mp-TiO_2_/bl-TiO_2_/FTO substrate by two consecutive spin-coating steps at 1000 rpm for 15 s followed by 5000 rpm for 20 s with a ramping rate of 300 and 1300 rpm s^−1^, respectively. One milliliter of ether was dropped onto the spinning substrate 10 s after starting the second spin-coating stage. The perovskite-deposited substrate was then heat-treated on a hot plate at 150 °C for 10 min. For deposition of the organic hole transporting material, 2,2′,7,7′-tetrakis-(N,N-di-4-methoxyphenylamino)-9,9′-spirobifluorene (spiro-OMeTAD) (88 mg in 1 mL of chlorobenzene) was mixed with 7.5 µL of lithium bis(trifluoromethanesulfonyl)imide (Li-TFSI) solution in acetonitrile (170 mg mL^-1^) and 7.5 µL of 4-tert-butylpyridine (*t*BP). The hole-conducting material was spin-coated at 3000 rpm for 30 s on the perovskite/mp-TiO_2_/bl-TiO_2_/FTO. Finally, a gold layer was deposited on the hole conducting layer using a thermal evaporator.

### DFT calculation details

All calculations were performed in the framework of the spin-polarized density functional theory with the projector augmented wave (PAW) method^63^ using the Vienna ab-initio simulation package (VASP) code^64^. The exchange-correlation was considered using the generalized gradient approximation of Perdew, Burke, and Ernzerhof (PBE)^65^. The cut-off energy for the plane-wave basis set was 500 eV, and Monkhorst-Pack k-point mesh of 4 × 4 × 1 was used for all the slab structure of α-Fe_2_O_3_ (hematite). The ionic positions were relaxed until a force convergence of 0.01 eV A^−1^ was reached. Because of the strongly correlated 3d states in transition metal oxide systems, we used the GGA + U framework to modify the self-interaction^66^. The values of U-J of all the 3d metals were set to 4.2 eV for good agreement with the experimental band-gap of α-Fe_2_O_3_ (2.2 eV). The hexagonal unit cell of α-Fe_2_O_3_ was optimized with a layered anti-ferromagnetic (AFM) ordering. For pure α-Fe_2_O_3_ unit cell, the lattice parameters calculated within PBE + U were *a* = *b* = 5.07 Å and *c* = 13.88 Å, and they were consistent with the experimental values of *a* = 5.04 Å and *c* = 13.75 Å^67^. Each fully relaxed bulk structure of pristine and Ge-doped α-Fe_2_O_3_ was used to determine the lattice parameters of each (1 × 1) slab structure. A vacuum layer at least 12Å was used to minimize the interaction between the periodic surface along *z* axis. We focused on the surface reaction on (0001) α-Fe_2_O_3_ surface because it is one of the natural growth faces of hematite^68^. Dopant substitutions were made at both outermost Fe layers to consider the maximum doping effect on surface reactions and to remove the polarization from broken symmetry^54^. Hydrogen passivation was used to prevent the transfer of hydrogen atoms from the active site to the other surface oxygen. We passivated only one of the three surface oxygen atoms to minimize the hydrogen bonding that affects the reaction.

We considered the following OER mechanism with four elementary steps^69^.

1 $${{\rm{H}}}_{2}{\rm{O}}+\ast \to \ast \text{OH}+{{\rm{H}}}^{+}+{{\rm{e}}}^{-}$$

2 $$\ast \text{OH}\to \ast \text{O}+{\text{H}}^{\text{+}}\text{+}\,{\text{e}}^{-}$$

3 $${{\rm{H}}}_{2}{\rm{O}}+\ast {\rm{O}}\to \ast {\rm{OOH}}+{{\rm{H}}}^{+}+{{\rm{e}}}^{-}$$

4 $$\ast {\rm{OOH}}\to \ast +{{\rm{O}}}_{2}+{{\rm{H}}}^{+}+{{\rm{e}}}^{-}$$

The * represents chemisorption with the reactive sites on the surface. According to Rossemiesl et al.^69^, at standard conditions (pH = 0, *p* = 1 bar, *T* = 298 K), the reaction free energy(${\triangle {\rm{G}}}$) of each step is calculated as follows:

5 $${\triangle {\rm{G}}}_{{\rm{A}}}={\Delta {\rm{E}}}_{\ast {\rm{OH}}}+{\left(\triangle {\rm{ZPE}}-{\rm{T}}\triangle {\rm{S}}\right)}_{{\rm{A}}}-{\rm{e}}\cdot \Phi$$

6 $${\triangle {\rm{G}}}_{{\rm{B}}}={\Delta {\rm{E}}}_{\ast {\rm{O}}}-{\Delta {\rm{E}}}_{\ast {\rm{OH}}}+{\left(\triangle {\rm{ZPE}}-{\rm{T}}\triangle {\rm{S}}\right)}_{{\rm{B}}}-{\rm{e}}\cdot \Phi$$

7 $${\triangle {\rm{G}}}_{{\rm{C}}}={\Delta {\rm{E}}}_{\ast {\rm{OOH}}}-{\Delta {\rm{E}}}_{\ast {\rm{O}}}+{\left(\triangle {\rm{ZPE}}-{\rm{T}}\triangle {\rm{S}}\right)}_{{\rm{C}}}-{\rm{e}}\cdot \Phi$$

8 $${\triangle {\rm{G}}}_{{\rm{D}}}=4.92{\rm{eV}}-{\Delta {\rm{E}}}_{\ast {\rm{OOH}}}+{\left(\triangle {\rm{ZPE}}-{\rm{T}}\triangle {\rm{S}}\right)}_{{\rm{D}}}-{\rm{e}}\cdot \Phi$$

${\Delta {\rm{E}}}_{\ast {\rm{OH}}}$, ${\Delta {\rm{E}}}_{\ast {\rm{O}}}$ and ${\Delta {\rm{E}}}_{\ast {\rm{OOH}}}$ are the binding energies for the adsorption of OH, O, and OOH, respectively. ZPE is the zero-point energy and ${\rm{T}}\triangle {\rm{S}}$ is entropic contributions. $\Phi$ is the external potential. At the standard condition with $\Phi$ = 0, the highest free energy ($\triangle {\rm{G}}_{{\rm{max }}}$) is equal to reaction potential for electrochemical reaction potential and (${\triangle {\rm{G}}}_{{\rm{max }}}-1.23$) is equal to overpotential (η).

## Supplementary information

Supplementary Information

## Data Availability

The data that support the findings of this study will be made available upon request.

## Source data

Source Data
