# Supplementary material for: NiFeOx decorated Ge-hematite/perovskite for an efficient water splitting system
Source: Nat Commun. 2021 Jul 14;12:4309. doi: 10.1038/s41467-021-24428-7 (PMC8280122; doi:10.1038/s41467-021-24428-7)
Supplement: Supplementary file 1 — Supplementary Information [file 41467_2021_24428_MOESM1_ESM.pdf]

Supplementary Information

$NiFeO_x$ Decorated Ge-doped Hematite/Perovskite for an Efficient Water Splitting System

Ki-Yong Yoon[1], Juhyung Park[1], Minsu Jung[2], Sang-Geun Ji[1], Hosik Lee[1], Ji Hui Seo[1], Myung-Jun Kwak[1], Sang Il Seok[1], Jun Hee Lee[1], and Ji-Hyun Jang[1*]

[1]School of Energy and Chemical Engineering, Department of Energy Engineering, Ulsan National Institute of Science and Technology (UNIST), Ulsan, Republic of Korea.

[2]School of Chemical and Environmental Engineering, Dong-Eui University, Busan, Republic of Korea

Corresponding Author: Ji-Hyun Jang (clau@unist.ac.kr)

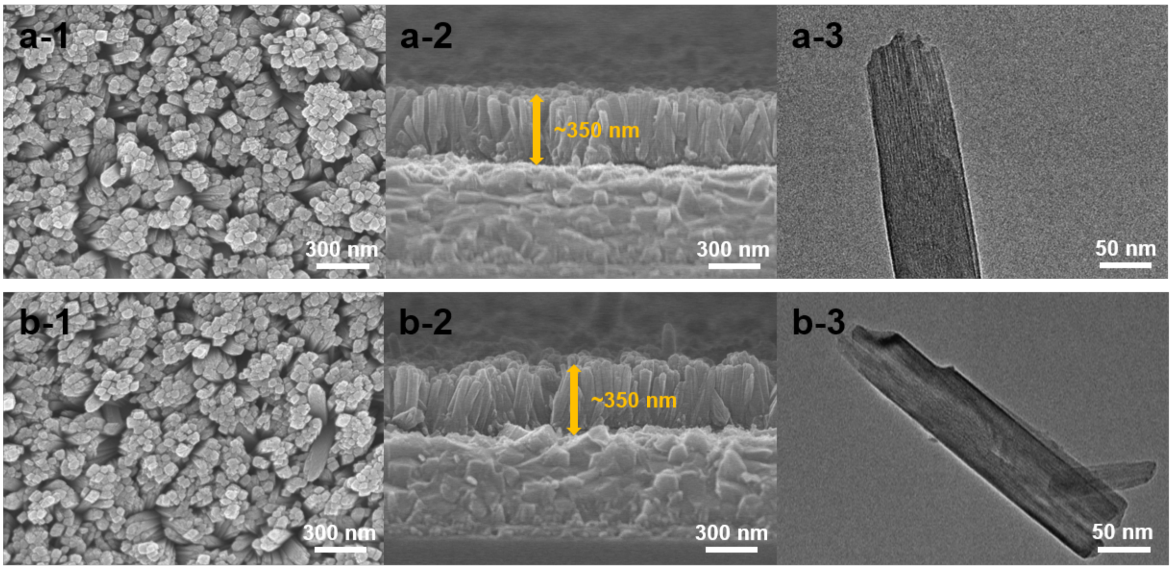

Supplementary Fig. 1 | SEM and TEM images of FeOOH and Ge doped FeOOH. The top-view (a-1 and b-1) and cross-sectional (a-2 and b-2) SEM images and a TEM image (a-3 and b-3) of **a,** FeOOH nanorod and **b,** Ge-doped FeOOH nanorod.

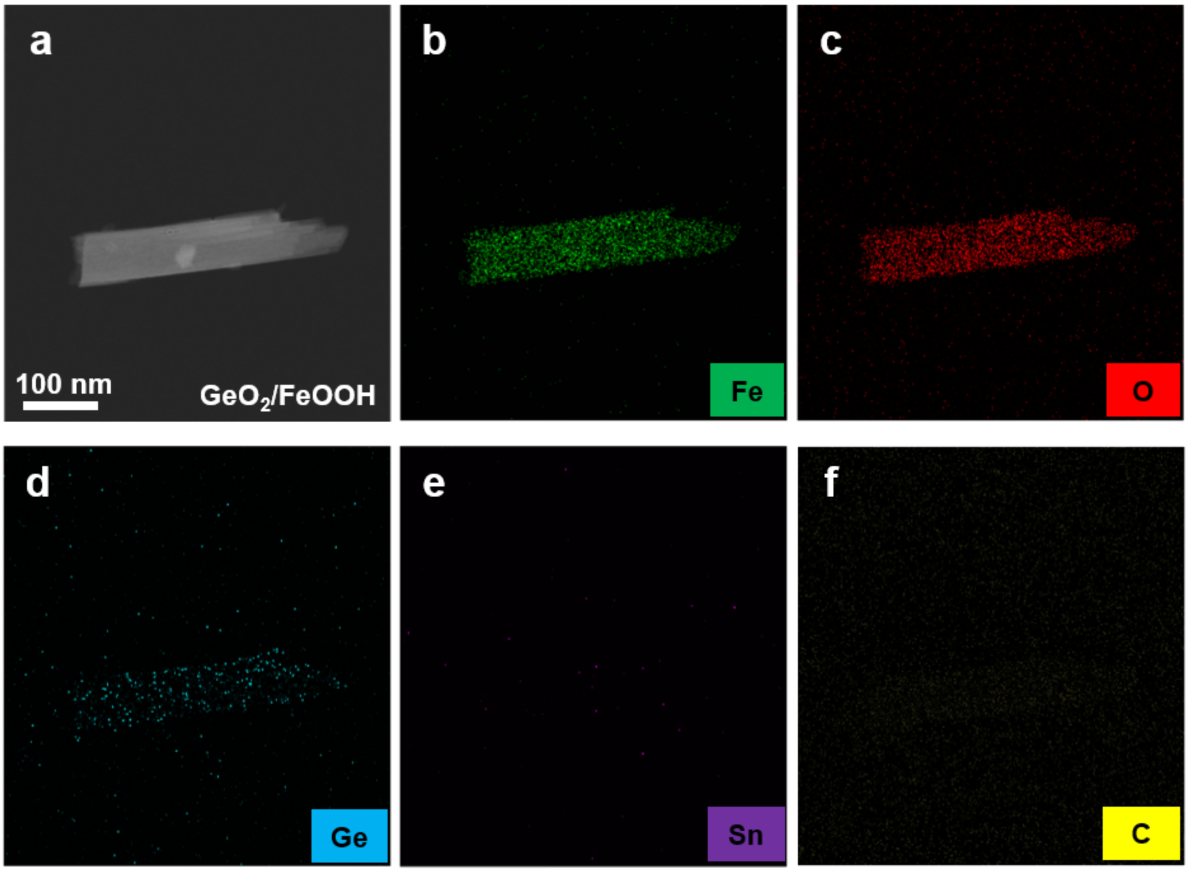

Supplementary Fig. 2 | TEM image of GeO_2/FeOOH. a, STEM image of the entire GeO_2/FeOOH nanorod and the corresponding elemental mapping image of **b,** Fe, **c,** O, **d,** Ge, **e,** Sn and **f,** C.

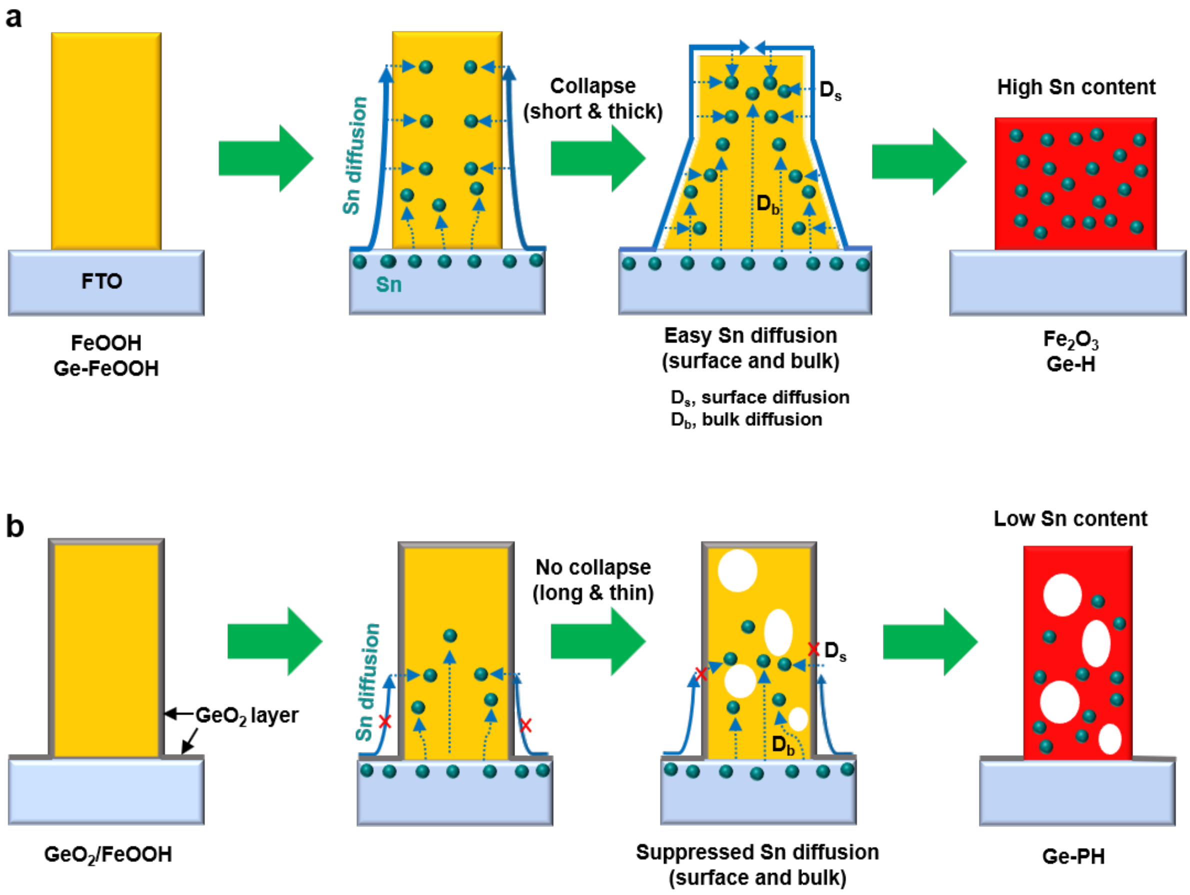

Supplementary Fig. 3 | Sn diffusion process. Illustration of Sn diffusion **a,** without the GeO_2 layer (Fe_2O_3 and Ge-H) and **b,** with the GeO_2 layer (Ge-PH).

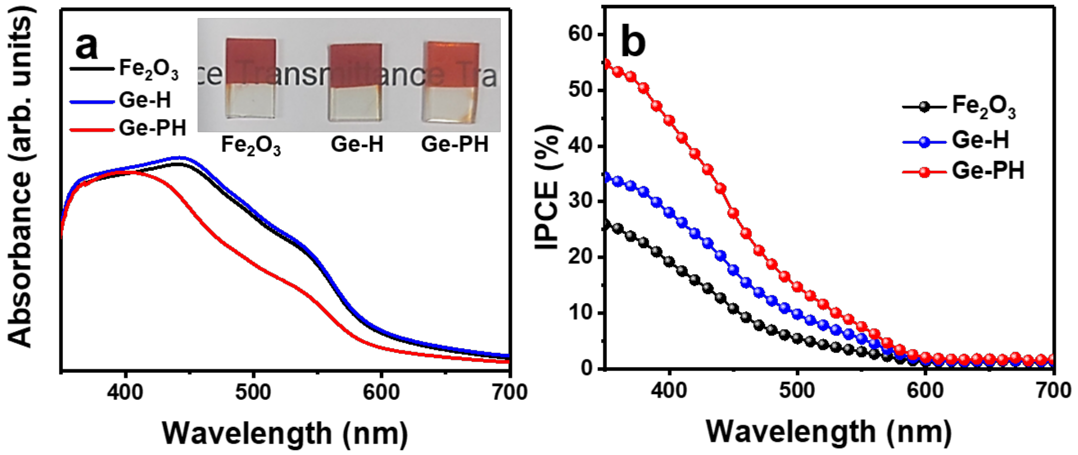

Supplementary Fig. 4 | a, UV-Vis and **b,** IPCE spectra of Fe_2O_3, Ge-H and Ge-PH.

Ge-H and Fe_2O_3 exhibited similar absorption in the range of 350-700 nm. However, Ge-PH showed lower absorption than Fe_2O_3 and Ge-H as shown in Supplementary Fig. 4a. This was because Ge-PH has porous structures. As can be seen in the digital camera image of the actual samples with similar thicknesses, Ge-PH is more transparent than the others due to the low density of the active material. The higher PEC performance of Ge-PH compared to Fe_2O_3 and Ge-H may be due to two strong advantages which compensate for the lower absorption. As shown in Supplementary Fig. 4b, Ge-PH has an outstanding incident-photon-to-current efficiency (IPCE) spectrum at all wavelengths over Fe_2O_3 and Ge-H despite the lowest UV-Vis absorption values. There are two main reasons for this result.

1) Shortened path length and sufficient active area due to porous morphology

Ge-PH in the form of nanoporous structures has a shortened hole diffusion pathway (~ 15 nm), which is beneficial for hematite with a short hole diffusion length (~5nm). In addition, the larger active area provides more reactive sites for OER reactions,

resulting in higher PEC performance.

2) Efficient doping

Our doping strategy is a way to maximize Ge doping efficiency by achieving much suppressed structural distortion in the surface region. This leads to a much reduced recombination, better electrical conductivity, and thus higher PEC performance.

In summary, although Ge-PH fabricated by our doping method exhibited poorer absorption properties than Fe_2O_3 and Ge-H, this was compensated for by an increase in the active area and the improved electrical conductivity. Therefore, Ge-PH achieved a highly enhanced PEC performance. More importantly, this higher transmittance, while achieving higher performance, is strongly beneficial for the tandem system where the solar cell is positioned behind the photoanode.

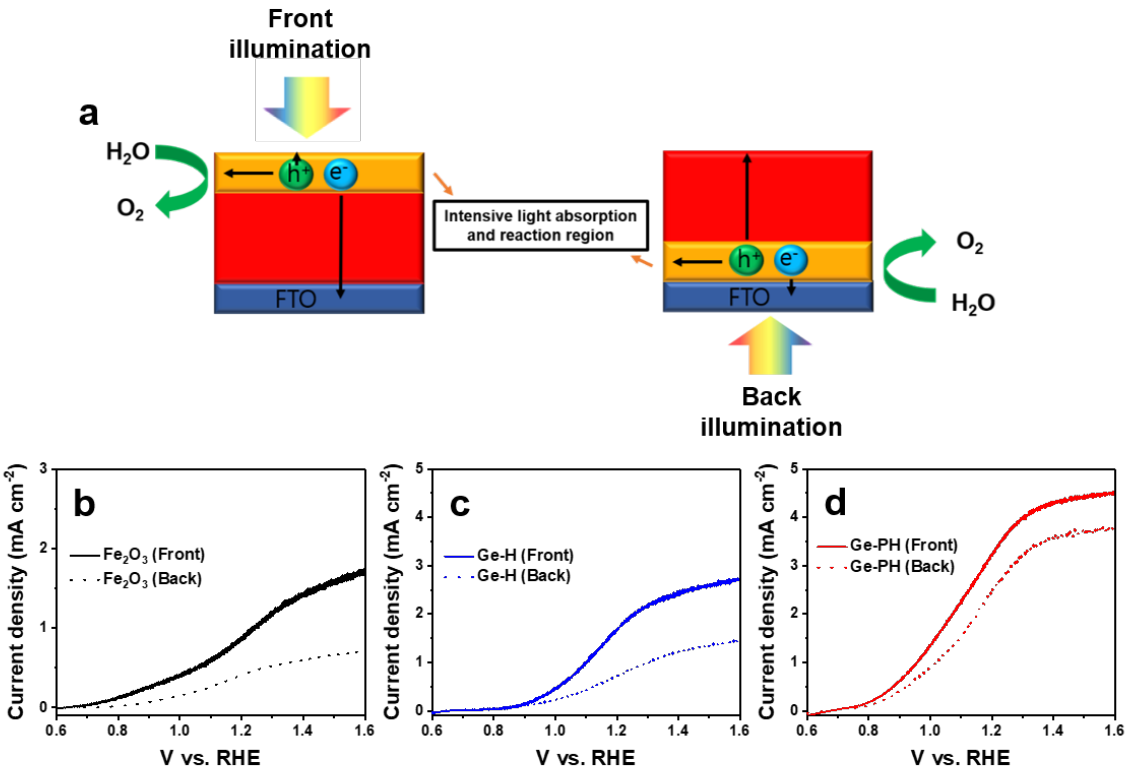

Supplementary Fig. 5 | The PEC performance by different light illumination. a, Schematic representation of electron/hole transport in a hematite photoanode under front (left) and backside (right) illumination. LSV curves of **b,** Fe_2O_3 **c,** Ge-H and **d,** Ge-PH under front and backside illumination.

We compared the PEC performance of the samples with front and back illumination, which proved that Ge-PH had much reduced structural distortion in the hematite lattice and the shorter diffusion length for holes to reach the surface due to the porous structure inside the nanorods.

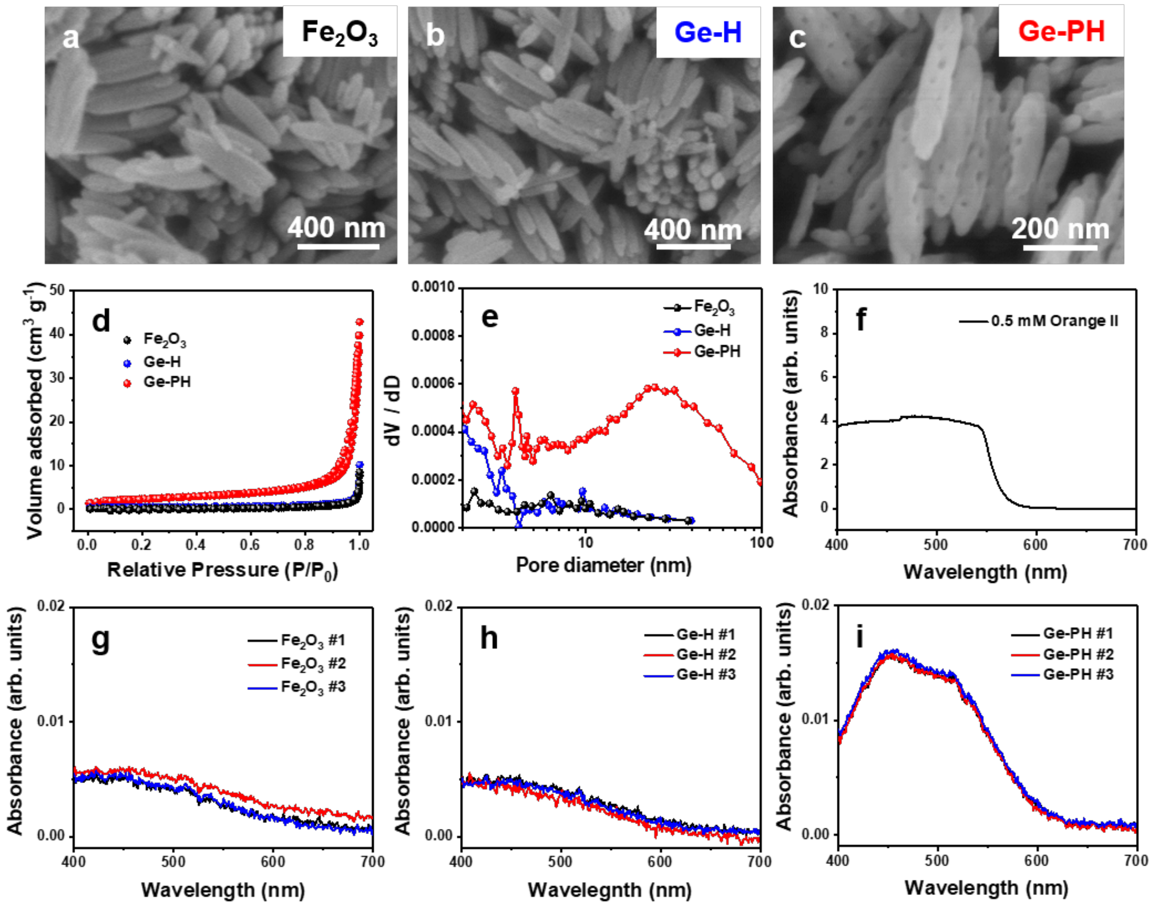

j	Real surface area (m^2)	Roughness factor
Fe_2O_3	0.001691±0.0001	11.27±1
Ge-H	0.001458±0.0001	9.72±1
Ge-PH	0.004924±0.0001	32.83±0.6

Supplementary Fig. 6 | BET analysis for surface area. SEM images of powder type hematite for BET measurements. **a,** Fe_2O_3, **b,** Ge-H and **c,** Ge-PH. **d,** N_2 adsorption-desorption isotherms of Fe_2O_3, Ge-H and Ge-PH. The BET surface area of each sample is around 2 m^2/g (Fe_2O_3 and Ge-H) and 10 m^2/g (Ge-PH). **e,** The pore distribution in Fe_2O_3, Ge-H and Ge-PH. UV-Vis absorbance of **f,** orange II dye, **g,** Fe_2O_3, **h,** Ge-H and **i,** Ge-PH. **j,** Calculated real surface area and the roughness factor of each sample.

For the BET measurement, we collected each precipitate prepared with the same process as the hematite nanorods on the FTO substrate to ensure sufficient quantity of the samples (0.5 g). The morphology of these precipitates is very similar to the nanostructure grown on the FTO substrate as shown in Supplementary Figs.6a-c. As shown in Supplementary Fig.6d, the surface area of porous hematite (Ge-PH, 10 m^2/g) is higher than that of nonporous hematite (Fe_2O_3 and Ge-H, 2 m^2/g). We also calculated the surface area and roughness factor of each sample by dye absorption as shown in Supplementary Figs. 6f-j. The roughness factor was calculated by the following equation:

$$\text{Roughness factor} = \frac{\text{Real surface area}}{\text{Geometric surface area}}$$

$$= \frac{\text{Desorbed dye x Avogadro number x Area of single dye}}{\text{Absorptivity x Path length x Geometric surface area}}$$

It was confirmed that the roughness factor of Fe_2O_3 and Ge-H were around 11.2 and 9.7 and the roughness factor of Ge-PH about 32.8, which was an increase of around three times.

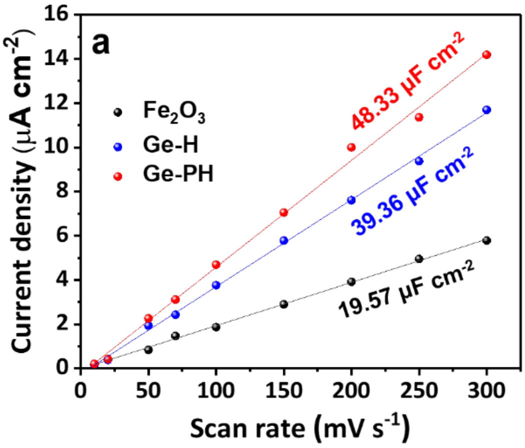

Supplementary Fig. 7 | The ECSA values of each photoanode.

The electrocatalytically active surface area (ECSA) values were obtained as follows:

$$ECSA = C_{dl}/C_s$$

where C_{dl} is the electrochemical double-layer capacitance of the catalytic surface, and C_s is the double layer capacitance of an atomically flat surface with 1 cm^2 of the real surface area under a 1M NaOH electrolyte condition.

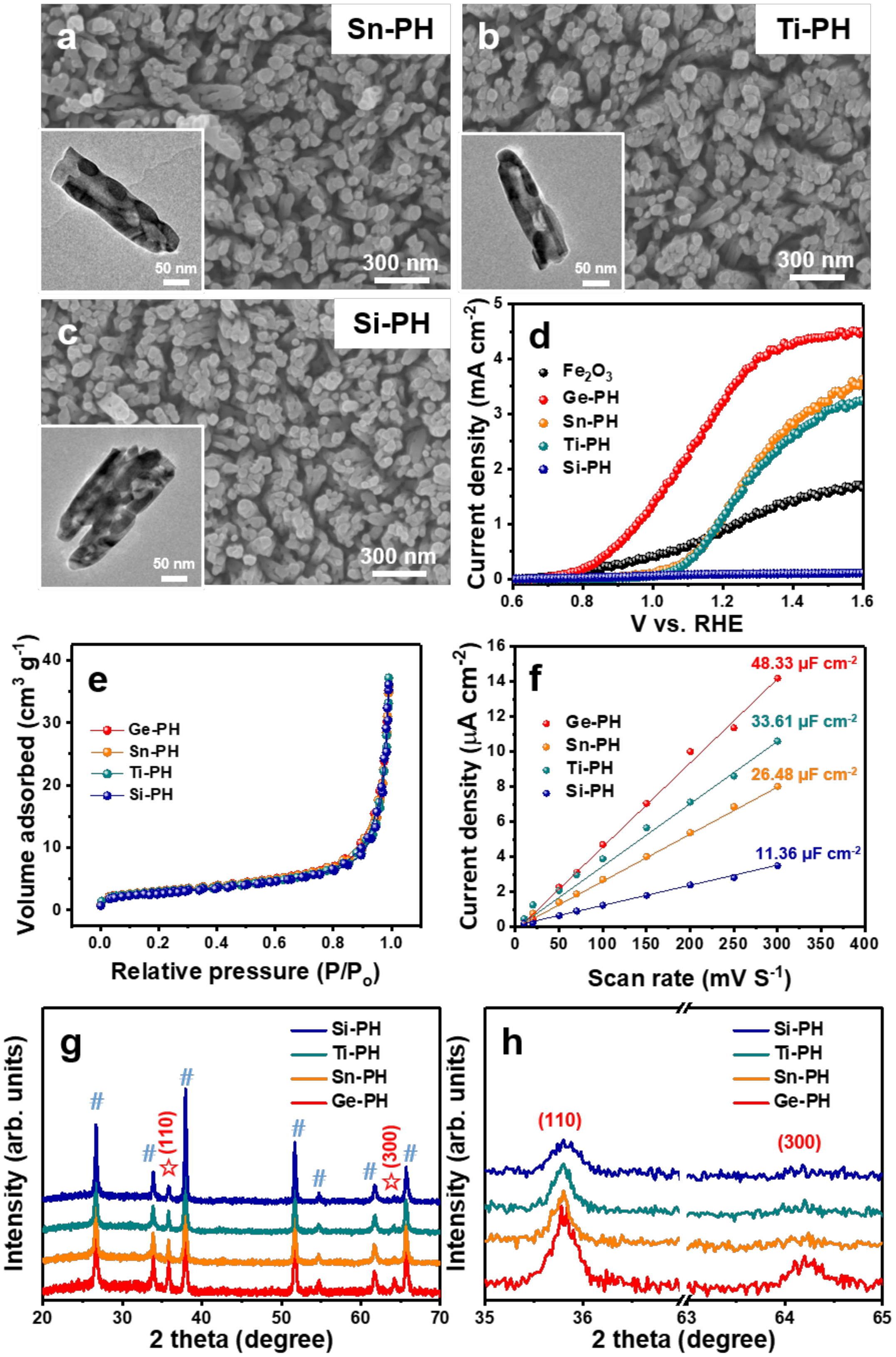

Supplementary Fig. 8 | PEC performance and hematite crystallinity of various

dopants in hematite. SEM images of **a**, intentional Sn doped porous hematite (Sn-PH) **b,** Ti-doped porous hematite (Ti-PH) and **c,** Si-doped porous hematite (Si-PH). The insets are TEM images for each sample. **d,** J-V curves of each photoanode. **e,** N_2 adsorption-desorption isotherms of Ge-PH, Sn-PH, Ti-PH and Si-PH. The BET surface area of each sample is around 10 m^2/g. **f,** The calculated ECSA values of each photoanode. **g,** XRD patterns of hematite with different dopants and **h,** the zoomed image in e showing the (110) and (300) plane.

We created a porous structure with intentional Sn, Ti, Si doping based on previous studies, by dipping in $SnCl_4$, $TiCl_4$ or APTMS solutions as shown in Supplementary Fig. 8a-c. Supplementary Fig. 8d shows that Ge-doped hematite had the best performance. In the case of Si-doping, particularly, the PEC performance decreased significantly.

In order to rule out that the improvement was due to the increased porosity, we have further conducted the BET measurement of the same porous hematite with different dopants (X-PH, where X=Si, Ti, Sn, and Ge).

As can be seen in Supplementary Fig. 8e, all samples (Si-PH, Sn-PH, Ti-PH, Ge-PH) exhibited similar BET results (around 10-11 m^3/g: Si-PH (10.8 m^3/g), Sn-PH (10.9 m^3/g), Ti-PH (10.5m^3/g), Ge-PH (10.7 m^3/g)) but different electrocatalytically active surface area (ECSA) values as shown in Supplementary Fig. 8f. This clearly showed the dopant effect (dopability) of heteroatoms into hematite. ECSA analysis considered both the surface area and the surface activity with the electrolyte induced by doping. Basically, a large surface area can positively contribute to the PEC performance, but it may vary according to different surface activities, which can be altered into different ECSA values. As an extreme example, although Si-PH had a larger surface area than pristine Fe_2O_3 (10.8 m^3/g vs 2 m^3/g), the ESCA value of Si-PH was much lower than

that of pristine Fe_2O_3 (11.36 $\mu F/cm^{-2}$ vs 19.57 $\mu F/cm^{-2}$), leading to a lower PEC performance. This indeed indicates the surface activity caused by the dopant effect (dopability of Si) primarily governed the overall performance rather than the surface area itself. Since silicon has a very high formation energy for doping in hematite, successful doping has only been achieved via a non-equilibrium process[2], where most Si-PH prepared in solution-based methods exhibited poor Si-dopability, which implies a close relation between dopability (doping effect) and PEC performance.

This is consistent with the results for crystallinity shown in Supplementary Figs. 8g and 8h. Although most peaks of hematite's crystallinity were overlapped by the peaks of FTO substrate, it can be seen that Ge-doped hematite has the best crystallinity in the (110) and (300) plane. This well matches the calculated formation energies for dopant solubility in hematite.

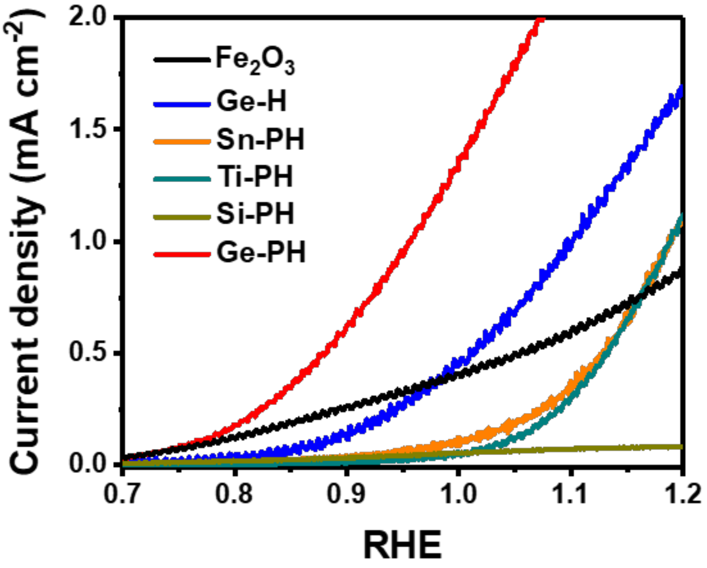

Supplementary Fig. 9 | Onset potential of various photoanodes in our experiments.

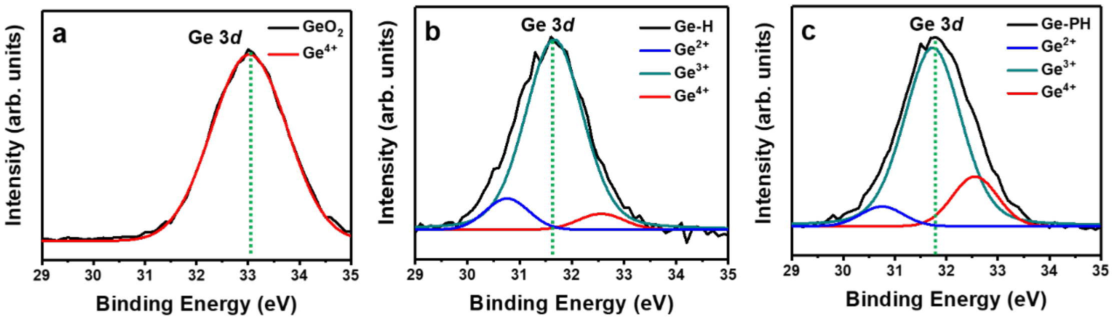

Supplementary Fig. 10 | XPS spectra of Ge 3d for **a,** GeO_2, **b,** Ge-H and **c,** Ge-PH.

GeO_2 showed the sharp Ge^{4+} peak only whereas Ge-H or Ge-PH exhibited various peaks with different valences in Ge 3d. It has been reported that the binding energy of Ge 3d for Ge-doped samples is about 31.7 eV, which is smaller than that of GeO_2 (33.2 eV)[1]. The binding energy of Ge 3d for Ge-doped samples slightly varies according to the doping level of Ge, where the one with a higher content of the Ge dopant is slightly larger than that with the lower doping level due to the charge transfer between the doping cation and the host atom (Fe^{3+}).

As can be seen in our XPS data, Ge-PH exhibits higher binding energy (31.8 eV) compared to Ge-H (31.6 eV) due to the higher content of doped Ge. This agrees well with the previous report[1].

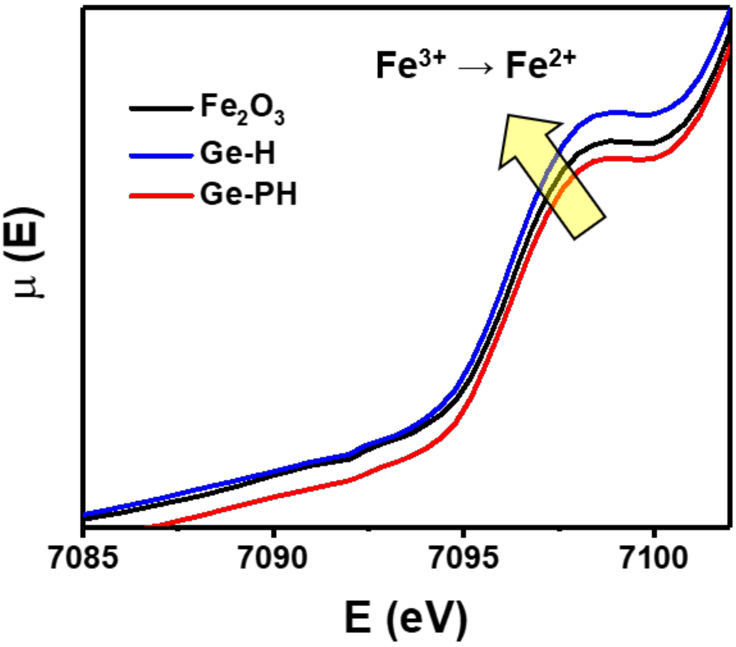

Supplementary Fig. 11 | XANES spectra of Fe_2O_3, Ge-H and Ge-PH.

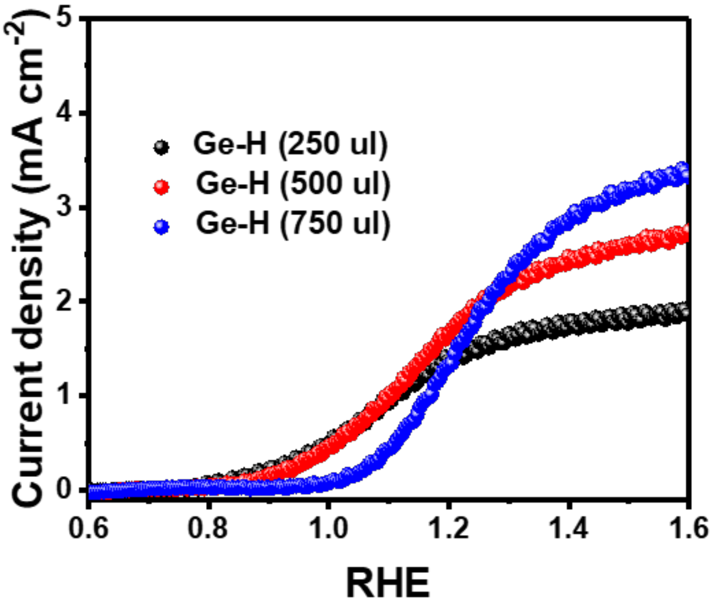

Supplementary Fig. 12 | The photoelectrochemical performance of Ge-H according to the amount of different Ge precursors in 100 ml of 150 mM $FeCl_3$.

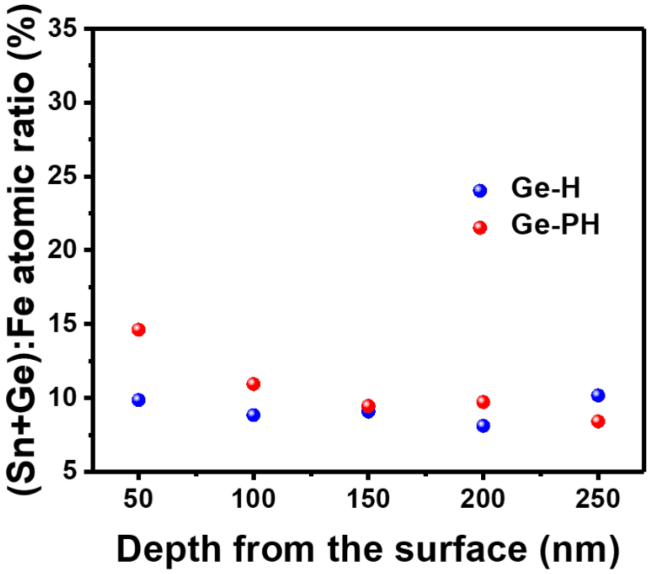

Supplementary Fig. 13 | The depth profiles of (Sn+Ge)/Fe ratio.

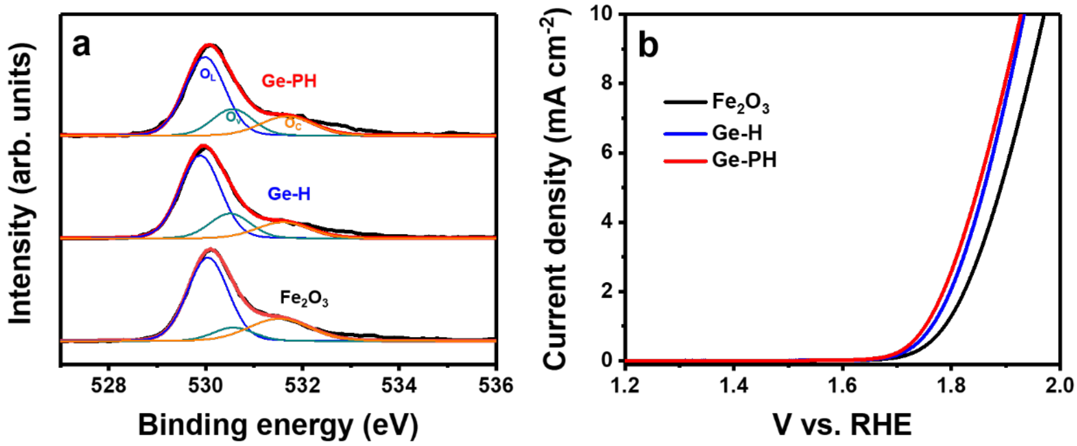

c	O_L (%)	O_C (%)	O_V (%)
Fe_2O_3	63.98	25.71	10.31
Ge-H	64.36	19.27	16.37
Ge-PH	59.14	20.84	20.02

Supplementary Fig. 14 | a, XPS spectrum of O 1s for Fe_2O_3, Ge-H and Ge-PH. **b,** LSV curves in the dark condition for OER. **c,** Table for comparison of O_L, $O_{C,}$ and O_V. The value is the area ratio of each peak in the XPS spectrum of the samples.

By deconvoluting O 1s signals, the oxygen species were characterized by binding energies at around 532.2, 531.9, and 533.3 eV for all samples, corresponding to the O^{2-} species in the hematite lattice (O_L), hydroxyl groups (OH^-) bonded to the metal cations (Fe-OH) in the oxygen deficient region (O_v), and chemisorbed or dissociated oxygen species from the H_2O molecules (O_c), respectively, as shown Supplementary Figs. 14a. The content of O_v obtained from the area ratio for the O 1s peak in Ge-PH was higher (20.02%) than that in Ge-H (16.37%) and in Fe_2O_3 (10.31%), indicating the abundant oxygen vacancies in the Ge-PH as shown in Supplementary Fig. 14c. All samples showed similar OER curves in dark conditions as shown in Supplementary Fig. 14b, which indicates that the level of the oxygen vacancy contained in our samples did not cause a change in the OER mechanism.

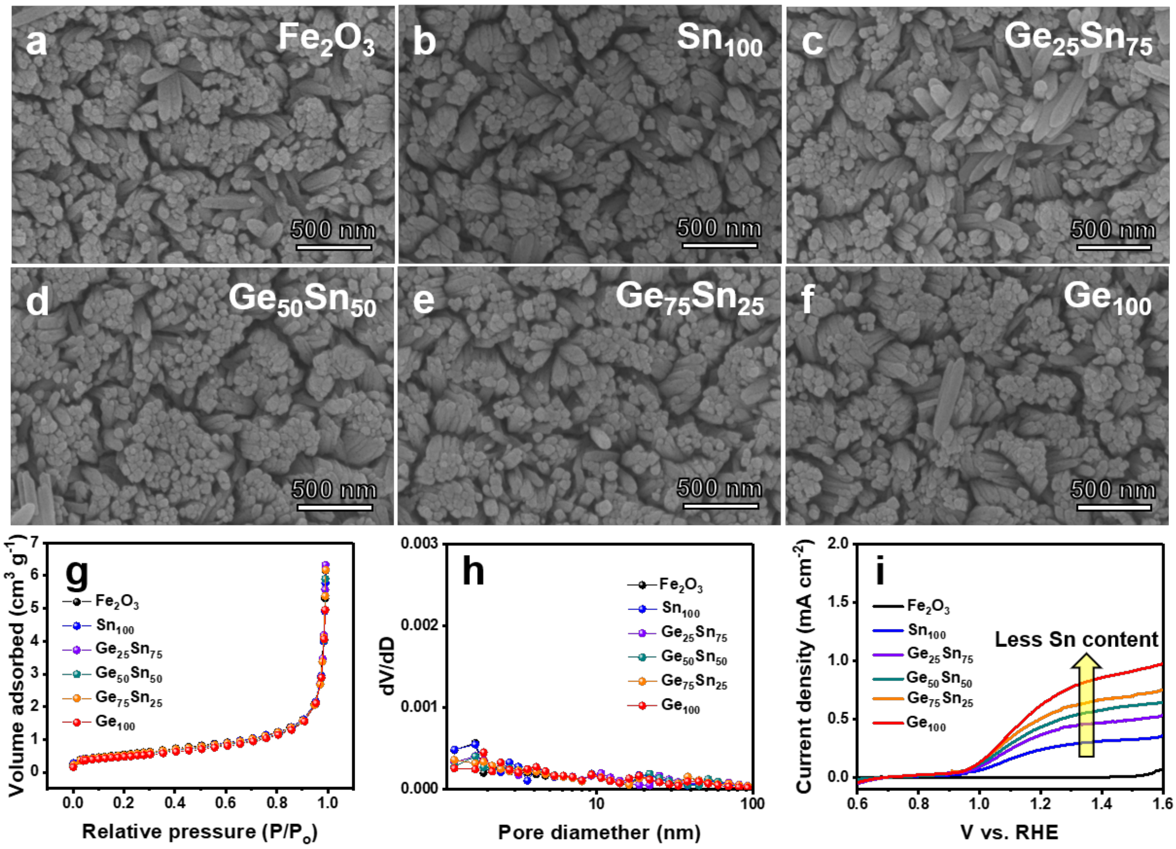

Supplementary Fig. 15 I SEM images of **a,** Fe_2O_3, **b,** Sn_{100} (Ge_0:Sn_{100}-hematite), **c,** $Ge_{25}Sn_{75}$ (Ge_{25}:Sn_{75}-hematite), **d,** $Ge_{50}Sn_{50}$ (Ge_{50}:Sn_{50}-hematite), **e,** $Ge_{75}Sn_{25}$ (Ge_{75}:Sn_{25}-hematite), **f,** Ge1$_{00}$ (Ge_0:Sn_{100}-hematite). **g,** N_2 adsorption-desorption isotherms of each sample. The BET surface area of each sample is around 2 m^2/g. **h,** The pore size distribution in Fe_2O_3, Ge-H, and Ge-PH. **i,** J-V curves of Sn-doped, Ge-doped, and Ge:Sn co-doped hematite prepared at low temperature (550 °C).

To exclude the Sn doping effect by thermal diffusion from the FTO substrate, we fabricated heteroatom-doped hematite at low temperature (550 °C). Each doping was performed in-situ by mixing a GeO_2 or $SnCl_4$ solution with a $FeCl_3$ solution. All samples did not exhibit micro/meso porosity as shown in Supplementary Figs. 15a-h. The surface area and porosity according to various doping conditions were almost identical. Therefore, the PEC performance depended primarily on the surface activity that was

differentiated by the concentration of each dopant. As shown in Supplementary Fig. 15i, as the Sn content increased, the Ge:Sn co-doping cases became worse, while the Ge single-doped sample showed the best performance, indicating that Ge is a better dopant than Sn.

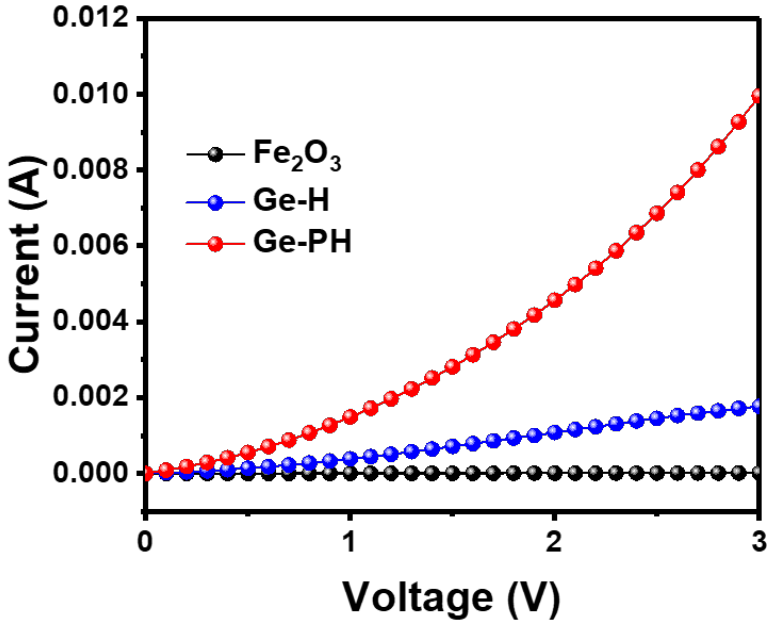

Supplementary Fig. 16 l Comparison of the electrical conductivity for each sample.

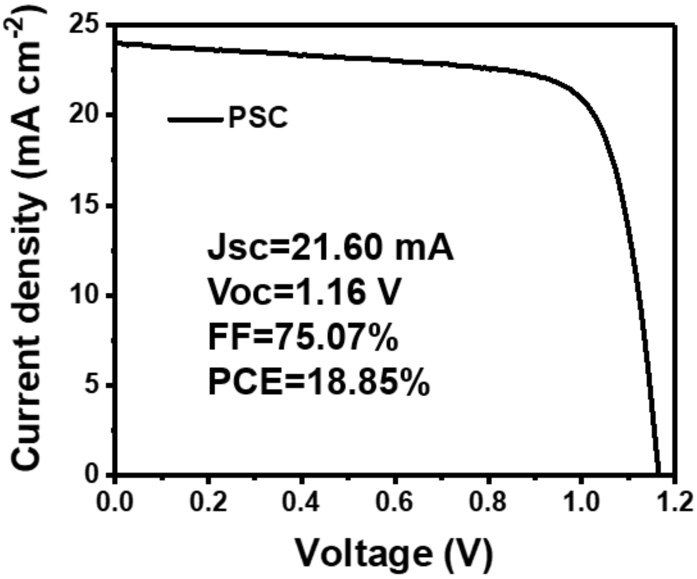

Supplementary Fig. 17 | J-V curve of perovskite solar cell under illumination.

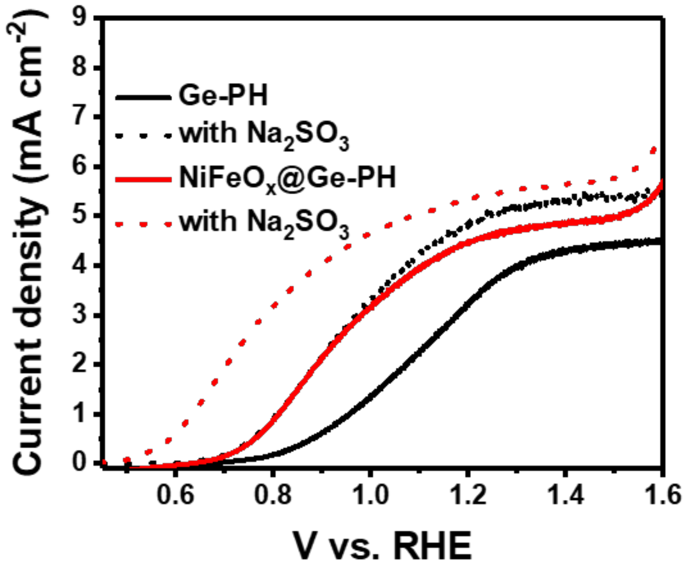

Supplementary Fig. 18 | The PEC performance for the $NiFeO_x$ catalyst with a hole scavenger.

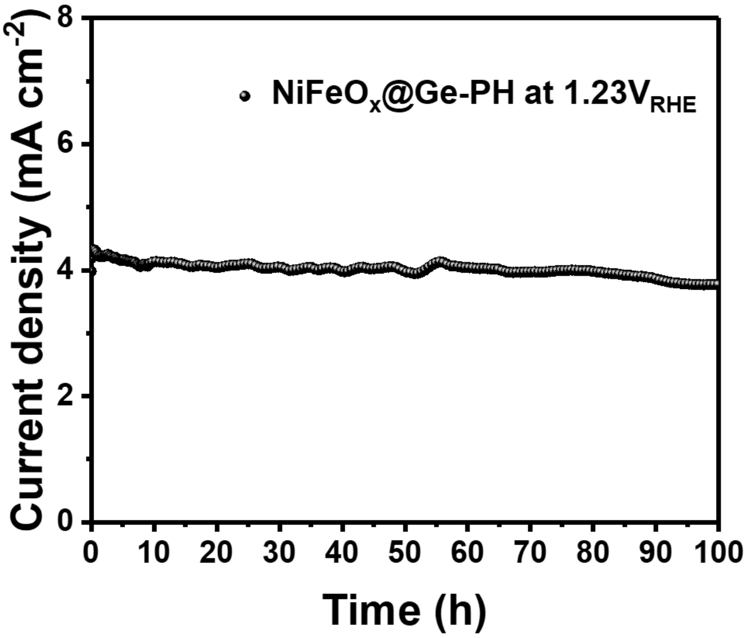

Supplementary Fig. 19 | Stability of $NiFeO_x$@Ge-PH at 1.23V_{RHE} for an extended period of time.

Supplementary Table 1. Recent reports on hematite-based photoanodes for surface area and doping concentration.

	Surface area (BET)	Surface area (ECSA)	Doping concentration	Photocurrent density ($1.23V_{RHE}$)	Reference
Mesoporous single crystals (MSCs) Fe_2O_3	18.2 $m^2\ g^{-1}$	-	-	~0.61 mA cm^{-2}	Nano Lett. (2015)[3]
Ti-Fe_2O_3	-	25.0 μF cm^{-2}	0.4%	~0.70 mA cm^{-2}	J. Mater. Chem. A (2017)[4]
F-Fe_2O_3	-	0.06 μF cm^{-2}	1.5%	~2.0 mA cm^{-2}	J. Mater. Chem. A (2018)[5]
F-Fe_2O_3	-	10.05 μF cm^{-2}	1.3%	~1.77 mA cm^{-2}	Nanoscale (2020)[6]
P-Fe_2O_3	-	229.1 μF cm^{-2}	5.9%	~0.78 mA cm^{-2}	Chem. Eng. J. (2019)[7]
**Fe_2O_3**<br>**Ge-H**<br>**Ge-PH**	**2 $m^2\ g^{-1}$**<br>**2 $m^2\ g^{-1}$**<br>**10 $m^2\ g^{-1}$**	**19.57 μF cm^{-2}**<br>**39.36 μF cm^{-2}**<br>**48.33 μF cm^{-2}**	**8.2%**<br>**9.6%**<br>**12%**	**1.0 mA cm^{-2}**<br>**1.9 mA cm^{-2}**<br>**3.5 mA cm^{-2}**	**This work**

Supplementary Table 2. Recent reports on hematite-based photoanodes with representative dopants (Si, Ti, Sn and Ge). The photocurrent density of all reports at $1.00V_{RHE}$ was confirmed approximately.

Dopant type	Photoanode	Measurement condition	Photocurrent density ($1.00V_{RHE}$)	Photocurrent density ($1.23V_{RHE}$)	Reference
Si	Si-Fe_2O_3	1 M NaOH (pH=13.6), 100 mW/cm^2	~0.30 mA/cm^2	~1.50 mA/cm^2	J. Am. Chem. Soc. (2006)[8]
Si	Co/ Si-Fe_2O_3	1 M NaOH (pH=13.6), 100 mW/cm^2	~1.00 mA/cm^2	~2.70 mA/cm^2	J. Am. Chem. Soc. (2006)[9]
Si	IrO_2/ Si-Fe_2O_3	1 M NaOH (pH=13.6), 100 mW/cm^2	~1.40 mA/cm^2	~3.01 mA/cm^2	Angew. Chem. Int. Ed. (2016)[10]
Si	$FeNiO_x$/ Al_2O_3/ Si-Fe_2O_3	1 M KOH (pH=13.6), 100 mW/cm^2	~1.50 mA/cm^2	~2.75 mA/cm^2	J. Am. Chem. Soc. (2015)[11]
Si, Ti	Si:Ti-Fe_2O_3	1 M NaOH (pH=13.6), 100 mW/cm^2	~0.10 mA/cm^2	~1.20 mA/cm^2	Appl. Phys. Lett. (2010)[12]
Ti	Co-Pi/ Ti-(SiO_x/np-Fe_2O_3)	1 M NaOH (pH=13.6), 100 mW/cm^2	~2.00 mA/cm^2	~3.19 mA/cm^2	Angew. Chem. Int. Ed. (2016)[13]
Ti	Ti-FeOOH/ Ti-PH	1 M NaOH (pH=13.6), 100 mW/cm^2	~2.10 mA/cm^2	~4.06 mA/cm^2	J. Mater. Chem. A (2016)[14]
Ti	Ni_2FeO_x/ H_2 treated TiO_2/ Ti-Fe_2O_3	1 M KCi (pH=9.2) or 1M NaOH (pH=13.6), 100 mW/cm^2	~2.30 mA/cm^2	~4.50 mA/cm^2	Nat. Commun. (2016)[15]
Ti	Co/ H_2 treated TiO_2/ H_2 treated Ti-Fe_2O_3	1M NaOH (pH=13.6), 100 mW/cm^2	~4.30 mA/cm^2	~6.00 mA/cm^2	Nano Energy (2017)[16]
Ti	$NiFe(OH)_x$/ Ti-Fe_2O_3	1 M KOH (pH=13.6), 100 mW/cm^2	~0.60 mA/cm^2	~2.30 mA/cm^2	ACS Appl. Energy Mater. (2018)[17]
Ti	$NiFeO_x$/ Ti-Fe_2O_3 NB	0.1 M KOH, 100 mW/cm^2	~1.00 mA/cm^2	~3.41 mA/cm^2	Small (2019)[18]
Ti	Co-Pi/ Ti-Fe_2O_3 MC	1M NaOH (pH=13.6), 100 mW/cm^2	~1.50 mA/cm^2	~3.50 mA/cm^2	Nat. Commun. (2019)[19]
Ti, Sn	Ti:Sn-Fe_2O_3 /TiO_2	1M NaOH (pH=13.6), 100 mW/cm^2	~0.25 mA/cm^2	~1.00 mA/cm^2	ACS Sustainable Chem. Eng. (2019)[20]
Ti, Si	$NiFeO_x$/ Ti-Fe_2O_3 NB (Dual photoanode)	1M NaOH (pH=13.6), 100 mW/cm^2	~2.70 mA/cm^2	~4.00 mA/cm^2	Nano Energy (2020)[21]
Sn	Sn-Fe_2O_3	1M NaOH (pH=13.6), 100 mW/cm^2	~0.50 mA/cm^2	~1.86 mA/cm^2	Nano Lett. (2011)[22]
Sn	Co/ Sn-Fe_2O_3	1M NaOH (pH=13.6), 100 mW/cm^2	~1.00 mA/cm^2	~2.80 mA/cm^2	J. Mater. Chem. A (2015)[23]
Sn	Co-Pi/ Sn-Fe_2O_3	1M KOH (pH=13.6), 100 mW/cm^2	~0.50 mA/cm^2	~2.20 mA/cm^2	Nano Lett. (2011)[24]
Sn	Co-Pi/ Sn-Fe_2O_3	1M NaOH (pH=13.6), 100 mW/cm^2	~2.00 mA/cm^2	~3.90 mA/cm^2	ChemSusChem (2011)[25]
Sn	Sn-Fe_2O_3 /SiMWs	1M NaOH (pH=13.6), 100 mW/cm^2	~2.50 mA/cm^2	~3.12 mA/cm^2	J. Mater. Chem. A (2018)[26]
Ge	Ge-Fe_2O_3	1M NaOH (pH=13.6), 100 mW/cm^2	~0.02 mA/cm^2	~0.03 mA/cm^2	J. Phys. Chem. C (2012)[27]
Ge	Ge-Fe_2O_3	1M NaOH (pH=13.6), 100 mW/cm^2	~0.05 mA/cm^2	~0.11 mA/cm^2	Nano Energy (2013)[28]
Ge	Ge-Fe_2O_3	1M NaOH (pH=13.6), 100 mW/cm^2	~0.20 mA/cm^2	~1.40 mA/cm^2	Nano Energy (2014)[29]
Ge	Ge-Fe_2O_3	1M NaOH (pH=13.6), 100 mW/cm^2	~0.20 mA/cm^2	~0.92 mA/cm^2	Int. J. Hydrog. Energy (2018)[30]
**Ge**	**$NiFeO_x$/ Ge-PH**	**1M NaOH (pH=13.6), 100 mW/cm^2**	**~3.17 mA/cm^2**	**~4.60 mA/cm^2**	**This work**

Supplementary Table 3. Recent reports on hematite-based photoanodes for tandem systems.

Photoanode	Measurement condition	Photocurrent density ($1.23V_{RHE}$)	In tandem with	J_{op}	STH conversion efficiency	Reference
$NiFeO_x$/ Fe_2O_3	Phosphate buffered solution (pH=11.8), 100 mW/cm^2	~1.40 mA/cm^2	a-Si:H/TiO_2/Pt	~0.74 mA/cm^2	~0.9%	Nat. Commun. (2015)[31]
TiO_2/ Co-Fe_2O_3	0.5 M Na_2CO_3 (pH=11.2), 100 mW/cm^2	~3.04 mA/cm^2	PSC	~0.50 mA/cm^2	~0.6%	RSC Adv. (2018)[32]
$FeNiO_x$/ Al_2O_3/Si-Fe_2O_3	1 M KOH (pH=13.6), 100 mW/cm^2	~2.75 mA/cm^2	PSC	~1.54 mA/cm^2	~1.9%	J. Am. Chem. Soc. (2015)[11]
Co-Pi/ Mn-Fe_2O_3	1 M NaOH (pH=13.6), 100 mW/cm^2	~3.50 mA/cm^2	PSC	~1.93 mA/cm^2	~2.4%	Nano Lett. (2015)[33]
Co-Pi/ Sn-Fe_2O_3	1 M NaOH (pH=13.6), 100 mW/cm^2	~3.90 mA/cm^2	PSC	~2.80 mA/cm^2	~3.4%	ChemSusChem. (2017)[25]
FeNiOOH/ Fe_2TiO_5/ Fe_2O_3/ ITO	1 M NaOH (pH=13.6), 100 mW/cm^2	~2.20 mA/cm^2	a-Si:H/ μc-Si:H/ μc-Si:H	~1.10 mA/cm^2	~1.4%	ChemSusChem. (2019)[34]
$NiFeO_x$/ Ti:Si-Fe_2O_3 (Dual photoanode)	1 M NaOH (pH=13.6), 100 mW/cm^2	~4.00 mA/cm^2	PSC	~3.65 mA/cm^2	~4.5%	Nano Energy (2020)[21]
**$NiFeO_x$/ Ge-PH**	1 M NaOH (pH=13.6), 100 mW/cm^2	**~4.60 mA/cm^2**	**PSC**	**~3.90 mA/cm^2**	**~4.8%**	**This work**

References

1. Liu, J. *et al.* Ge-doped hematite nanosheets with tunable doping level, structure and improved photoelectrochemical performance. *Nano Energy.* **2**, 328-336 (2003).

2. Zhou, Z., Huo, P., Guo, L. & Prezhdo, O. V. Understanding hematite doping with group IV elements: a DFT+ U study. *J. Phys. Chem. C* **119**, 26303-26310 (2015).
3. Wang, C. W. *et al.* Engineered hematite mesoporous single crystals drive drastic enhancement in solar water splitting. *Nano Lett.* **16**, 427-433 (2016).
4. Fan, Z., Xu, Z., Yan, S. & Zou, Z. Tuning the ion permeability of an Al2O3 coating layer on Fe2O3 photoanodes for improved photoelectrochemical water oxidation. *J. Mater. Chem. A* **5**, 8402-8407 (2017).
5. Li, F. *et al.* Construction of an efficient hole migration pathway on hematite for efficient photoelectrochemical water oxidation. *J. Mater. Chem. A* **6**, 23478-23485 (2018).
6. Wang, C. *et al.* Activating a hematite nanorod photoanode via fluorine-doping and surface fluorination for enhanced oxygen evolution reaction. *Nanoscale* **12**, 3259-3266 (2020).
7. Bu, X., Gao, Y., Zhang, S. & Tian, Y. Amorphous cerium phosphate on P-doped Fe2O3 nanosheets for efficient photoelectrochemical water oxidation. *Chem. Eng. J.* **355**, 910-919 (2019).
8. Cesar, I., Kay, A., Martinez, J. A. G. & Grätzel, M. Translucent thin film Fe2O3 photoanodes for efficient water splitting by sunlight: nanostructure-directing effect of Si-doping. *J. Am. Chem. Soc.* **128**, 4582-4583 (2006).

9. Kay, A., Cesar, I. & Grätzel, M. New benchmark for water photooxidation by nanostructured α-Fe2O3 Films. *J. Am. Chem. Soc.* **128**, 15714-15721 (2006).

10. Tilley, S. D., Cornuz, M., Sivula, K. & Grätzel, M. Light-induced water splitting with hematite: improved nanostructure and iridium oxide catalysis. *Angew. Chem. Int. Ed.* **49**, 6405-6408 (2010).

11. Morales-Guio, CG. et al., An optically transparent iron nickel oxide catalyst for solar water splitting. *J. Am. Chem. Soc.* **137**, 9927-9936 (2015).

12. Zhang, M., Luo, W., Li, Z., Yu, T. & Zou, Z. Improved photoelectrochemical responses of Si, Ti codoped α-Fe2O3 photoanode films. *Appl. Phys. Lett.* **97**, 042105 (2010).

13. Ahn, H.-J., Yoon, K. Y., Kwak, M. J. & Jang, J.-H. A Titanium-Doped SiOx Passivation Layer for Greatly Enhanced Performance of a Hematite-Based Photoelectrochemical System. *Angew. Chem. Int. Ed.* **55**, 9922-9926 (2016).

14. Yoon, K.-Y. *et al.* A selectively decorated Ti-FeOOH co-catalyst for a highly efficient porous hematite-based water splitting system. *J. Mater. Chem. A* **4**, 18730-18736 (2016).

15. Kim, J. H. *et al.* Hetero-type dual photoanodes for unbiased solar water splitting with extended light harvesting. *Nat. Commun.* **7**, 13380 (2016).

16. Jeon, T. H., Moon, G.-H., Park, H. & Choi, W. Ultra-efficient and durable photoelectrochemical water oxidation using elaborately designed hematite nanorod arrays. *Nano Energy.* **39**, 211-218 (2017).

17. Li, M. *et al.* Boosting the photoelectrochemical Water Oxidation at hematite photoanode by innovating a hierarchical ball-on-wire-array structure. *ACS Appl. Energy Mater.* **1**, 5836-5841 (2018).

18. Tang, S. *et al.* 3D hierarchical nanorod@nanobowl array photoanode with a tunable light-trapping cutoff and bottom-selective field enhancement for efficient solar water splitting. *Small* **15**, 1804976 (2019).

19. Zhang, Z. *et al.* Interfacial oxygen vacancies yielding long-lived holes in hematite mesocrystal-based photoanodes. *Nat. Commun.* **10**, 4832 (2019).

20. Park, J. W. *et al.* Improved interfacial charge transfer dynamics and onset shift in nanostructured hematite photoanodes via efficient Ti4+/Sn4+ heterogeneous self-doping through controlled TiO2 underlayers. *ACS Sustainable Chem. Eng.* **7**, 6947-6958 (2019).

21. Park, J. *et al.* A highly transparent thin film hematite with multi-element dupability for an efficient unassisted water splitting system. *Nano Energy.* **76**, 105089 (2020).

22. Ling, Y., Wang, G., Wheeler, D. A., Zhang, J. Z. & Li, Y. Sn-doped hematite nanostructures for photoelectrochemical water splitting. *Nano Lett.* **11**, 2119-2125 (2017).

23. Qin DD. *et al.*, Sn-doped hematite films as photoanodes for efficient photoelectrochemical water oxidation. *J. Mater. Chem. A* **3**, 6751-6755 (2015).

24. Li., M. *et al.*, Morphology and doping engineering of Sn-doped hematite nanowire photoanodes. *Nano Lett.* **17**, 2490-2495 (2017).

25. Gurudayal *et al.*, Atomically altered hematite for highly efficient perovskite tandem water-splitting devices. *ChemSusChem.* **10**, 2449-2456 (2017).

26. Zhou. Z. *et al.*, Modulating oxygen vacancies in Sn-doped hematite film grown on silicon microwires for photoelectrochemical water oxidation. *J. Mater. Chem. A* **6**, 15593-15602 (2018).

27. Liu., J., Liang, C., Zhang, H., Tian, Z. & Zhang, S. General strategy for doping impurities (Ge, Si, Mn, Sn, Ti) in hematite nanocrystals. *J. Phys. Chem. C* **116**, 4986-4992 (2017).

28. Liu., J. *et al.*, Ge-doped hematite nanosheets with tunable doping level, structure and improved photoelectrochemical performance. *Nano Energy* **2**, 328-336 (2013).

29. Liu., J. *et al.*, Highly oriented Ge-doped hematite nanosheet arrays for photoelectrochemical water oxidation. *Nano Energy* **9**, 282-290 (2014).

30. Zhao, L. *et al.* Enhanced efficiency of hematite photoanode for water splitting with the doping of Ge. *Int. J. Hydrog. Energy* **43**, 12646-12652 (2018).

31. Jang, J.-W. *et al.*, Enabling unassisted solar water splitting by iron oxide and silicon. *Nat. Commun.* **6**, 7447 (2015).

32. Roy, S. & Botte G. G. Perovskite solar cell for photocatalytic water splitting with a TiO_2/Co-doped hematite electron transport bilayer. *RSC Adv.* **8**, 5388-5394 (2018).

33. Gurudayal *et al.*, Perovskite-hematite tandem cells for efficient overall solar

driven water splitting. *Nano Lett.* **15**, 3833-3839 (2015).

34. Urbain, F. *et al.*, Multilayered hematite nanowires with thin-film silicon photovoltaics in an all-earth-abundant hybrid tandem device for solar water splitting. *ChemSusChem.* **12**, 1428-1436 (2019).
